# Personal Branding: Interdisciplinary Systematic Review and Research Agenda

**DOI:** 10.3389/fpsyg.2018.02238

**Published:** 2018-11-21

**Authors:** Sergey Gorbatov, Svetlana N. Khapova, Evgenia I. Lysova

**Affiliations:** Department of Management and Organization, Vrije Universiteit Amsterdam, Amsterdam, Netherlands

**Keywords:** personal branding, personal brand, self-presentation, self-marketing, career

## Abstract

Personal branding has become an important concept in management literature in recent years. Yet, with more than 100 scholarly papers published on the concept to date, it has developed into a fragmented area of research with a diversity of definitions and conceptual boundaries. This paper posits that this heterogeneity of extant research impedes theoretical and empirical advancement. To strengthen the foundation for future work, we review the extant literature and offer an integrative model of personal branding. Through our systematic literature review we identify the key attributes of the construct, establish its clarity by comparing it with similar concepts in its nomological network, and suggest the definitions of *personal branding* and *personal brand* based on the reviewed literature. Further, we propose a theoretical model of personal branding summarizing the findings from the reviewed papers. The proposed model outlines the trends conducive to personal branding, as well as its drivers, processes, and outcomes. Finally, we discuss ethical implications of personal branding for both scholarly work and practice. In conclusion, we outline a further research agenda for studying personal branding as a critical career and organizational behavior activity in contemporary working environment.

## Introduction

Marketing-born and reared, personal branding has made its definitive headway into management science. Sitting at the junction of marketing, sociology, communication, psychology, organizational behavior, and some would claim even accounting (Vitberg, 2010), personal branding has emerged as a means of attaining career success in the context of more temporary employment systems and project based work structures.

Many reasons have prompted the emergence and penetration of the concept—personal branding—into the management discourse. Among the key is a widespread shift of the responsibility for employees' careers from organizations to individuals (Arthur and Rousseau, 1996; Arthur, 2014; Greenhaus and Kossek, 2014). Indeed, business changes in traditionally stable sectors push thousands of lifetime workers out of jobs, e.g., because of the “greening” of the energy sector, or massive job cuts in the call centers, and because of the advances in artificial intelligence. More frequent career transitions require expanding and creating new networks of contacts, which, in turn, predicate more frequent personal rebranding activities (Schlosser et al., 2017). With the technological advances bringing about the ease of communication across the Internet and numerous social media platforms, “careers have become personal brands that need to be managed in a virtual age” (Gioia et al., 2014). When Peters (1997) wrote that everyone is a CEO of his or her own company, it must have been prescient to the labor market situation of today, where careers are boundaryless (psychological contract wanes) (Arthur et al., 2005), individuals are as good as their last gig (portfolio careers) (Cawsey, 1995), and “you are your own enterprise” (the need to be intelligent in career decisions) (Arthur et al., 2017).

Although personal branding originated in the field of marketing (Lair et al., 2005), there are now more than a hundred published papers on the topic across a range of disciplines. These papers contribute to the growing body of literature that aims to define personal branding, explain how it works, and to conceptualize it in relation to various input and output variables. Yet, this body of literature is diverse and disconnected, without any attempt so far to bring scholarly efforts together toward a more integrated understanding. No commonly accepted academic definitions or theoretical models exist. As the voice of popular press on personal branding becomes increasingly pervasive, painting a consistent picture that standard work is obsolete, that self-fulfillment is a *sine qua non* of success, and that organizational and personal interests are diverging (Vallas and Cummins, 2015), science needs to step forward to corroborate or refute such allegations. With this literature review we aim to fill this gap.

We analyze 100 papers on personal branding published in journals representing various disciplines, with the purpose to, firstly, synthesize all definitions of personal branding stemming from different disciplines and fields of studies, and to propose a new definition that integrates multidisciplinary knowledge about the concept. Secondly, we establish the personal branding's construct clarity, by positioning personal branding as a distinct construct alongside other established concepts related to managing perceptions of others toward achieving a specific objective, such as image, fame, or self-promotion. Thirdly, we propose a conceptual model of personal branding based on the reviewed literature outlining successive inputs, processes and outputs. Finally, a future research agenda is laid out by positioning personal branding as one of the essential human activities for maintaining sustainable work and employment.

## Methodology

**Figure 1:**
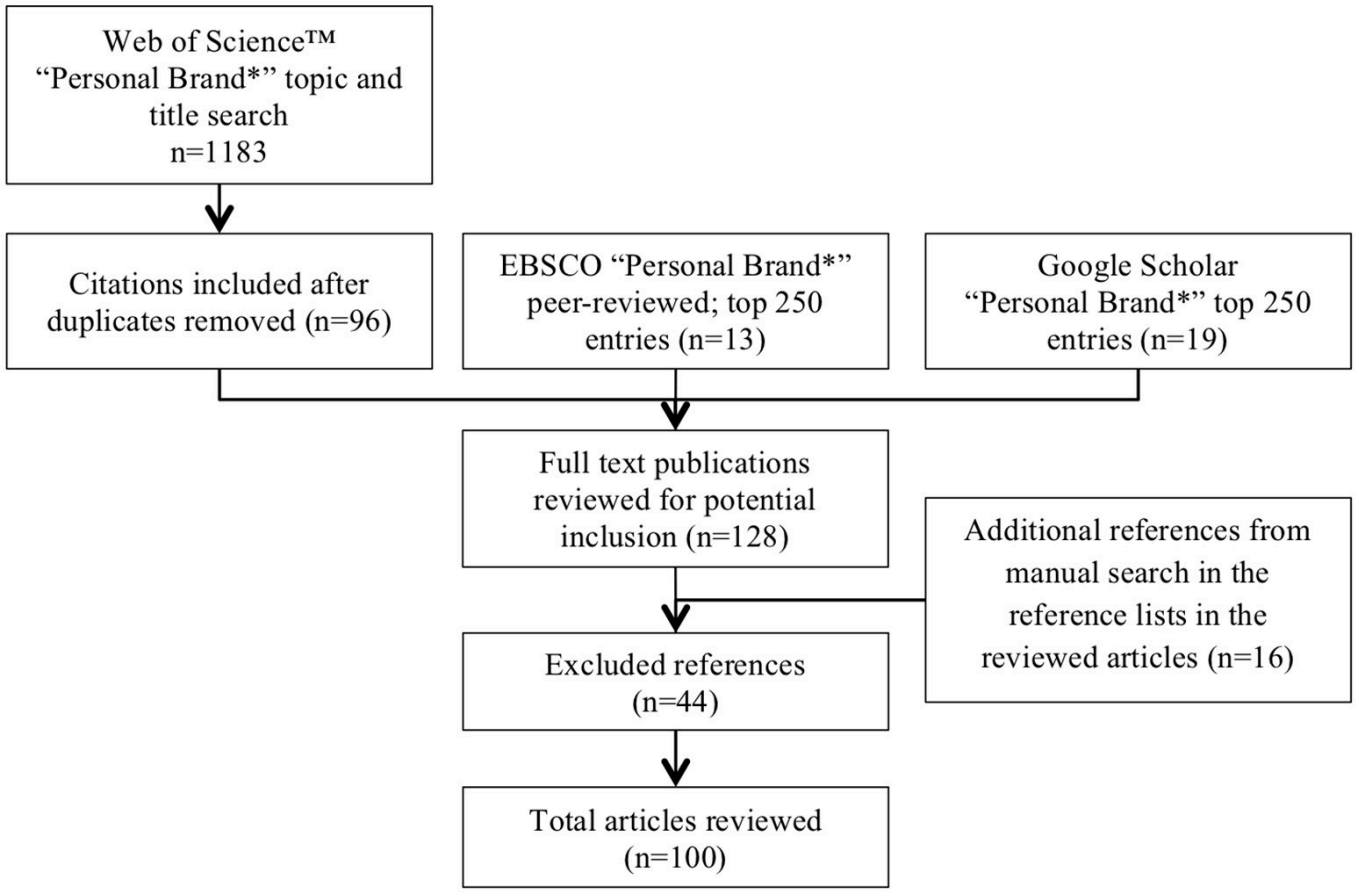
Review methodology process.

This field of knowledge being fragmented and scarce, we conducted a systematic literature review, applying wide criteria to include all the extant academic research on personal branding. A systematic approach intends to remove subjectivity and bring about cohesion through the synthesis of available information. To ensure a comprehensive approach and minimize the bias, where applicable, we followed the PRISMA guidelines for systematic reviews, suggested by Moher et al. (2009), related to defining the research question, setting the search parameters, extracting and appraising the relevant data, and synthesizing the findings. We followed the literature selection process used by Mol et al. (2015), followed by the “snowballing” technique (Greenhalgh and Peacock, 2005). An initial search by topic and title on Web of Science™ on April 1, 2018 returned 1183 results from all databases after applying the following restrictions: TOPIC OR TITLE: (personal brand^*^), Refined by: Research Domains: (Social Sciences OR Arts Humanities) AND Document Types: (Article OR Review) AND Research Areas: (Business Economics OR Psychology OR Communication OR Social Sciences Other Topics OR Sociology), Timespan: All years, Search language = Auto. Most of the articles in the topic search were related to the marketing studies of product branding, and, therefore, were excluded, as they were not relevant to the research topic of personal branding. Similarly, we did not consider non-academic papers and patents. Removing the duplicates across the topic and title search and studying the abstracts, 96 references were selected for full-text analysis. To ensure that any unindexed references are included, additional Boolean searches on the keywords “personal brand^*^” were carried out on EBSCO Business Source Complete restricting it by peer-reviewed publications only and on Google Scholar, returning 13 and 19 additional original references respectively; top 250 hits were manually reviewed in each search. After analyzing the full texts of the articles, 44 references were excluded for the following reasons: (a) for lacking academic rigor albeit published in peer-reviewed journals (*N* = 16), (b) for lacking relevance to the topic of the study (*N* = 14), and (c) for being in a language that the researcher did not know (*N* = 10), and for the inability to find full text articles (*N* = 4). A manual search in the reference lists of the selected articled resulted in 16 additional references added to the list. Conference proceedings and papers were included. As a result, this current review is based on the analysis of full text of 100 academic publications. This process is graphically explained in Figure 1. Each article was subsequently analyzed in depth with the results coded under the corresponding category titles, main ones being definition, theory, model, methods, population, inputs, processes, outputs, study design, primary social media, future research recommendations.

**Figure 2:**
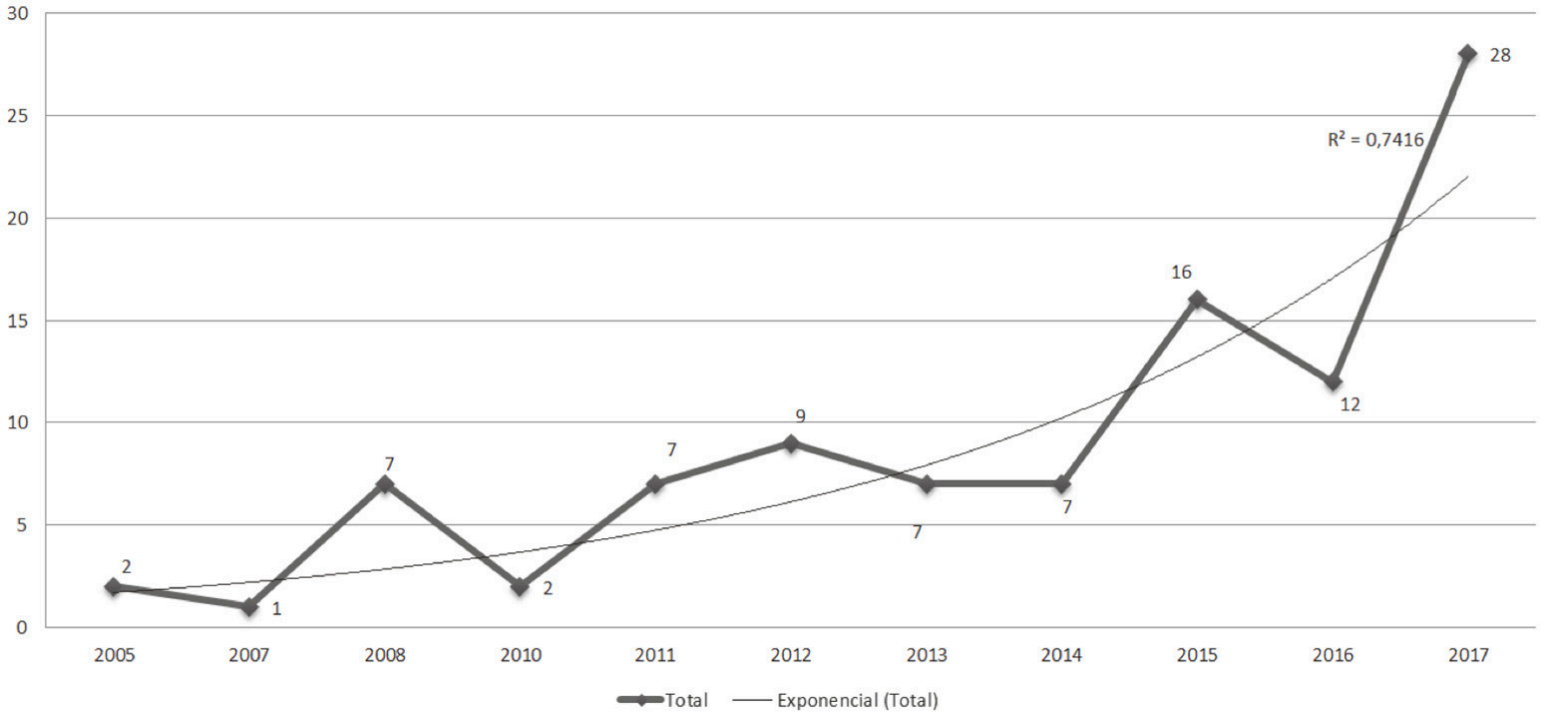
Total number of academic publications (*n* = 98) by year included in current review. The 2018 publications (*n* = 2) are excluded from this graph to prevent distortion of the exponential trend line, as the year is not over yet.

Considering that the first academic papers on the topic were published in 2005, the review period for this paper was set as 2005–2017. Since 2005, there has been an uptake in scholarly writing on the subject, and the growths in academic research and writing on the topic of personal branding follows an exponential trend line (*R*2 = 0.7416) as illustrated in Figure 2.

## Findings

As our review reveals, research on personal branding is progressively moving from conceptualization to empirical studies, with a preference for qualitative methods. Out of the 100 reviewed papers, 34 are conceptual. 42 papers used qualitative methods, 17—quantitative, and seven used a mixed-method approach. Supplementary Table 1 lists all the reviewed papers in chronological order, together with the definition of *personal branding* or a *personal brand*, the nature of conducted research and the populations studied.

### Construct clarity and definitions personal branding

Looking through the literature, we found that despite a substantial number of academic articles on the topic of personal branding suggesting a diversity of definitions, there is little agreement on the exact boundaries of the concept. Therefore, as the first step, it deems necessary to determine the construct clarity and position it in the field of related concepts. Then, we elucidate the definitions of *personal branding* and *personal brand*, clearly demarcating them as self-standing constructs. We conclude this part with the analysis of the theoretical premises for personal branding that the earlier authors based their research in.

#### Construct clarity

While the authorship of the term “personal branding” in 1997 is contended by Montoya and Vandehey (2002) and Peters (1997), some researchers indicate the origins of the concept either in Goffman's work in 1960s (Lorgnier and O'Rourke, 2011; Khedher, 2015; Philbrick and Cleveland, 2015) or in the 1980s in marketing studies (Vallas and Cummins, 2015). Despite these early attempts, the academic work to research personal branding as a self-standing concept only began in early 2000s.

Shepherd (2005) reviewed the popular literature on the subject and acknowledged wide acceptance of the term “personal branding.” Some researchers use the term “self-branding” (Gandini, 2016), which is synonymous to personal branding. Still, this review finds that the term “personal branding” is more customary and accepted. Parmentier et al. (2013) made an attempt at the conceptual rapprochement among different definitions, stating that despite various names “the premise of much of what has been written is that some product branding concepts are sufficient for understanding how people can position themselves to be successful in any career pursuit” (p. 373). We hope to contribute further to greater construct clarity for personal branding. In order to do so, we followed the process suggested by Podsakoff et al. (2016). We present our findings in the next four sub-sections: (1) analysis of the definitions encountered in the reviewed literature; (2) study of the related concepts in the nomological network of personal branding as informed by this literature; (3) synthesis of the key attributes of personal branding from the reviewed definitions and analyzing presence or absence of the identified attributes in the related concepts; and (4) defining *personal branding* and a *personal brand*.

#### Heterogeneity of extant definitions

Although the definitions encountered in the studied articles are diverse, they can be grouped according to the underlying theoretical approach. We have identified two main categories of those definitions: Those based in the marketing theory and those sprouting from the studies of self-presentation behaviors. The “marketing” definitions (see, for example, Lair et al., 2005; Marwick and boyd, 2011; Bendisch et al., 2013) tend to use words like “product,” “buyer,” “seller,” “market,” “added value,” “promise,” “differentiation,” or “meeting customer needs.” They liken personal branding to a product branding process, using similar terminology and directly applying marketing principles. The “self-presentation” definitions (see, for example, Parmentier et al., 2013; Molyneux, 2015; Schlosser et al., 2017) tend to include such words as “impression,” “reputation,” “individual's strengths,” “uniqueness,” “image,” “self-promotion,” or “identity.” These definitions position personal branding as a person-centric activity, focused on managing how others view the individual. Although some papers use the definitions suggested by other scholars, there is no commonly accepted way to define personal branding in either approach. Also, we find that the existing definitions, provided in Supplementary Table 1, lack either in comprehensiveness, e.g., “active process of synthesizing and packaging a personal brand to target customers, prospective employers, and an online network of colleagues” (Cederberg, 2017, p. 1), rigor, e.g., “planned process in which people make efforts to market themselves” (Khedher, 2015, p. 20), or both, e.g., “how we want to be perceived by employers, potential employers, clients, professional peers, and others in a way that will boost short- and long-term career prospects” (Evans, 2017, pp. 271–272).

#### Related concepts

There are seven related concepts, chosen for this exercise, as they were consistently mentioned alongside with *personal branding* in the reviewed literature. They belong to the same group for the reason that they deal with perceptions of others of an individual. However, the agency of managing those perceptions, the vector of action, the nature of methods and techniques, and their intent are different, which gives way to distinguishing them one from the others. Zinko and Rubin (2015) in their work on personal reputation have provided a useful overview of several concepts under consideration, including reputation, status, image, fame, celebrity, pedigree, legitimacy, credibility, branding, and impression management. In our study, we have chosen the following most relevant seven related concepts with their definitions, as they were most frequently mentioned in relation to personal branding:

- ***Human branding***. Close et al. (2011) defined human brand as “persona, well-known or emerging, who are the subject of marketing, interpersonal, or inter-organizational communications” (p. 923). This concept comes from marketing, building upon the branding literature and extending it from products to people (Thomson, 2006).
- ***Impression management***. Kowalski and Leary (1990) defined impression management as “the process by which individuals attempt to control the impressions others form of them” (p. 34). It is the “vehicle by which professional image construction occurs” (Roberts, 2005).
- ***Self-promotion***. While Molyneux (2015) placed an equation mark between personal branding and self-promotion, we would like to disambiguate the two. Bolino et al. (2016) view self-promotion as a distinct impression management technique, when actors “are inclined to highlight their accomplishments, take credit for positive outcomes, name-drop important others, and downplay the severity of negative events to which they are connected” (p. 384).
- ***Image***. Roberts (2005) provided an authoritative point of view on professional image, also influencing our understanding of personal branding in considering the desired and perceived components of the personal brand (see further section on *Brand Architecture*). Yet, we would like to extract the “professional” part from her definition, given that image construction may occur outside of the organizational setting, so that it becomes “the aggregate of key constituents' < …> perceptions of one's competence and character” (p. 687).
- ***Reputation***. Several authors liken reputation to a personal brand (Noble et al., 2010; Schlosser et al., 2017), yet there are distinct differences between these concepts. Zinko and Rubin (2015), noting that the research on reputation is not yet well-developed, propose their own definition of it: “a perceptual identity formed from the collective perceptions of others, which is reflective of the complex combination of salient personal characteristics and accomplishments, demonstrated behavior, and intended images presented over some period of time as observed directly and/or reported from secondary sources, which reduces ambiguity about expected future behavior” (p. 218). While we would disagree with the word “intended” in this definition, as reputations can be formed in the most unintended manners, this is the most robust one we have found.
- ***Fame***. Zinko and Rubin (2015) suggested that fame equals reputation less predictability, since fame can be brought about by singular events, and later developed into reputation through repeated behavioral displays.
- ***Employee Branding***. While not often mentioned in the literature on personal branding, this concept is very close to the one under study, differing only in few key attributes. Miles and Mangold (2004) conceptualized employee branding within the framework of internal marketing, and defined it as “the process by which employees internalize the desired brand image and are motivated to project the image to customers and other organizational constituents” (p. 68).

#### Clarifying the construct of personal branding: key attributes

We will now proceed to the discussion of each of the five first-level attributes (strategic, positive, promise, person-centric, and artifactual), which were drawn from the definitions found in the reviewed literature.

##### Strategic

Several definitions used in the reviewed literature specifically point out that personal branded activities are *targeted*, i.e., directed at a defined audience (Labrecque et al., 2011; Cederberg, 2017), and *programmatic*, i.e., designed as a series of coordinated activities (Lair et al., 2005; Manai and Holmlund, 2015). There are some definitions using the word strategically directly (Marwick and boyd, 2011; Kleppinger and Cain, 2015; Nolan, 2015; Lee and Cavanaugh, 2016). For certain roles, strategic personal branding is a prerequisite. For example, Bendisch et al. (2013) discussed closing the gap between the desired identity, image, and reputation for CEO brands from the stakeholder and organizational perspectives, requiring a planful and deliberate approach. Gandini (2016), studying digital freelance professionals in London and Milan, likens strategic personal branding to a profitable form of investment of time, labor, and relationships, essential in a reputation economy. Such concepts as “fame” actively lack these characteristics, and they are not essential for “self-promotion,” “reputation,” or “image.” Bolino et al. (2016) note that while impression management can be strategic and intentional, it also can be “unconscious and habitual” (p. 378), hence we conclude that the programmatic aspect of impression management may be missing.

##### Positive

The definitions of personal branding are consistent in the positive intentionality of personal branding. Authors concur that its main objective is to “establish favorable impressions” (Lee and Cavanaugh, 2016), be “appealing” (Omojola, 2008), and “valuable, reliable or desirable” (De la Morena Taboada, 2014). We use the term “positive” as “desired by the target audience,” as indeed, there may be cases where personal branders would want to be associated with characteristics that are in ill regard by the societal norms, such as in research of male sex workers by Phua and Caras (2008). From this perspective, we can argue that “positive” also could be “drawing attention,” following the line of reasoning that one of the objectives of personal branding is to differentiate oneself in the emerging attention economy (Hearn, 2008b). The inability to create a positive desired image in the minds of the target audience or a mismatch between the goal and perception is a branding failure. Labrecque et al. (2011) identified two types of personal branding failures: *Insufficient branding* (e.g., lack of content, failure to emphasize the desired message, etc.) and *misdirected branding* (e.g., inconsistencies with the brand identity, addressing wrong audiences, etc.). They offer specific advice to increase the positive attribute of a personal brand: “Reinforcement for optimal branding, augmentation for insufficient branding, and deleting or diffusing for misdirected branding” (p. 47).

##### Promise

The marketing nature of the personal branding construct implies the idea of signaling a promise to the target audience (Tulchinsky, 2011; Philbrick and Cleveland, 2015). Parmentier et al. (2013), studying positioning of personal branding in the organizational field of modeling, concluded that effective signaling of one's human, social, and cultural capital depends on successfully fitting into a specific organizational field (*cf*. product brand points of parity) and standing out from the competition in that field (*cf*. product brand points of differentiation). In contrast to product brands, standing out in personal branding is achieved not by having additional attributes or characteristics but having higher levels of those qualities, valued by the target audience. The most adjacent concepts related to this attribute are human branding (Thomson, 2006) and employee branding (Miles and Mangold, 2004), both of which are built on the foundational purpose of a *brand* to convey a promise. Human branding is a generic concept, which may lack agency in cases when, for instance, an advertising agency brands a movie character, rather than the actor playing that character. Employer branding lacks reflexivity as that work is conducted top-down, guided by the overall organizational objectives.

##### Person-centric

This attributes comprises three second-level attributes: agency, reflective, and differentiation. The principle of agency supposes an active involvement of the subject of personal branding into the process: “Workers are encouraged to view themselves as entrepreneurs within corporate employment or while seeking corporate employment” (Lair et al., 2005, p. 316). While human branding, employee branding, fame, and reputation may occur without the subject's volition, personal branding demands the individual's involvement. Since personal branding requires agency and intentionality, persistent claims that “everybody has a personal brand” (Rampersad, 2008, p.34) are misguided, calling for a more accurate “everybody has a reputation.” Reflexivity highlights the exteriorization processes that are central to personal branding, where the subjects are required to identify individual characteristics prior to engaging in positioning of their personal brands to the outer world (Wee and Brooks, 2010). We have already highlighted that human branding and employee branding may lack reflexivity as an attribute due to low agency. Finally, differentiation refers to building a personal brand around a set of characteristics that are unique and desirable by the target audience (Parmentier et al., 2013). Studying personal branding of professional golfers, Hodge and Walker (2015) discuss how differentiation, or “standing out” from the competition, allowed those sportsmen to access valuable career opportunities.

##### Artifactual

Both personal branding and core marketing literature points out artifactual nature of branding. Examples of artifacts in personal branding go back to embroidering monograms on shirts, personalized stationary and visiting cards, or a signature at the bottom of a painting. Khedher (2015) specifically attributes artifactual displays of impression management behaviors to personal branding activities. Scholars are unanimous regarding the need for a *narrative* (Brooks and Anumudu, 2016; Eagar and Dann, 2016; Pera et al., 2016) and related *imagery* (van der Land et al., 2016; Holton and Molyneux, 2017). Several papers specifically studied the artifacts of personal branding efforts, such as narrated selfies (Eagar and Dann, 2016), LinkedIn photos (van der Land et al., 2016), Instagram photos (Geurin-Eagleman and Burch, 2016), YouTube videos (Chen, 2013), and ePortfolios (Jones and Leverenz, 2017). Concepts like reputation or impression management do not necessarily require a coherent story or associated artifacts.

Juxtaposing the identified attributes with other related concepts, we determine these attributes necessary and sufficient (Podsakoff et al., 2016) to demarcate the construct of personal branding as self-standing and distinct. The overview of the attributes of personal branding, compared to related concepts, is depicted in Table 1.

**Table 1:** Attributes of personal branding compared to related concepts.

**First-level attributes**	**Strategic**		**Positive**	**Promise**	**Person-centric**			**Artifactual**	
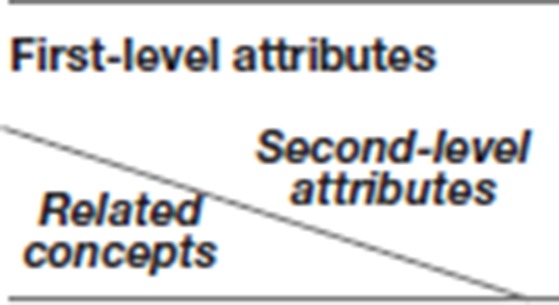		**Targeted**	**Programmatic**	**Always Positive**	**Promise**	**Agency**	**Reflexive**	**Differentiation**	**Narrative**	**Imagery**
Personal branding	P	P	P	P	P	P	P	P	P
Human branding	P	P	P	P		A	P	P	P
Impression management	P		P		P			
Self-promotion	P		P		P			
Image				A	P		P		P
Reputation							P	P
Fame	A	A		A		A	P	P	P
Employee branding	P	P	P	P		A	P	P	P

*Legend: P, present; A, absent (if blank, presence or absence of an attribute is situation-dependent)*.

#### Defining personal branding

Having identified the core attributes of the construct in question, we proceeded to elucidating its definition. Guided by the characteristics of a “good definition” (Suddaby, 2010), we propose the following way to define *personal branding*:

*Personal branding is a strategic process of creating, positioning, and maintaining a positive impression of oneself, based in a unique combination of individual characteristics, which signal a certain promise to the target audience through a differentiated narrative and imagery*.

In the reviewed literature, the authors would choose to base their work either on the definition of personal branding as a process, or a personal brand as a product, or both. Hence, we offer a definition of a personal brand as well. Drawing on the definition of personal branding and one provided by Ottovordemgentschenfelde (2017), we proceed to define a *personal brand*:

*Personal brand is a set of characteristics of an individual (attributes, values, beliefs, etc.) rendered into the differentiated narrative and imagery with the intent of establishing a competitive advantage in the minds of the target audience*.

### Theoretical foundations of personal branding

Personal branding, being a multidisciplinary construct, employs a wide range of distinct theories to explain it. We have grouped the theories used in the reviewed literature into four large categories: sociological, marketing, psychological, and economic.

#### Sociological theories

The majority of the authors, totaling 38 papers, used sociological theories to explain the concept of personal branding. Goffman's (1959) dramaturgical perspective is most often referenced (19 papers), positioning personal branding as both a backstage activity (e.g., reflection, sense-making, etc.) and onstage performance (impression management, feedback-seeking, etc.) to influence the perceptions of others. Meyrowitz (1990) extended the dramaturgical theory into wider social and digital contexts (cited by one paper). While Goffman's work on self-presentation and social interactions is a predominant way to understand the activities around personal branding, it does not explain fully the interactions in the digital world, and it may overlook some ways to understand the outcomes of personal branding.

As an extension to Goffman's work, specific research on impression management by Kowalski and Leary (1990), Baumeister (1982), Gardner and Martinko (1988), and Schlenker (1980) was mentioned in three papers. Linked to the backstage activities, four papers rely on the reflexivity theories of Giddens (1991), Beck (1992) and Adams (2003, 2006) attempt to explain how individuals build own identities in the fast-changing technological world. Five papers used Bourdieu's (1993) theories to explain accumulation of social and cultural capital in specific organizational fields, highlighting that our identities are shaped by the habitus and we are not in full control over them. Finally, Du Gay's enterprising culture theory (Gay and Salaman, 1992; Du Gay, 1996) is used in six papers to position personal branding as a new type of labor in the post-Fordist era, working identities forged into “enterprising selves” or “flexible subjectivities.”

#### Marketing theories

Shepherd (2005) noted that Kotler was first to expand the field of marketing beyond the product. Hughes (2007), Neale et al. (2008), and Speed et al. (2015) attributed the emergence of personal branding as a separate discipline to Keller's distinguishing the “small b” approach to branding, referring to product branding only, and the “large b,” extending the science of branding to services, organizations, and people. The work of Aaker (1997) on brand personality and brand identity is most often referenced in research on personal branding (seven papers). Thomson (2006) contributed to the stream of thinking around human brands. Eagar and Dann (2016) suggest three approaches to the self as a human brand: (1) “consumerist”—viewing human brands from the position of consumers, (2) “reputational”—assuming a passive approach in having a brand, and (3) “agency”—proactively creating and managing one's personal brand. An overwhelming majority of the extant literature on personal branding subscribes to the latter two approaches: understanding the brand equity, or the reputation, and managing the desired projected image. Overall, marketing theories were used in 17 papers.

#### Psychological theories

Eleven papers used psychological theories to explain personal branding. Four papers (Shepherd, 2005; Gioia et al., 2014; Molyneux, 2015; Holton and Molyneux, 2017; Schlosser et al., 2017) highlight the role of personal branding in identity formation, situating their thinking in the works of Mead (1934), Erikson (1968), Turner and Oakes (1986), Ibarra (1999), and others. Schlosser et al. (2017) even likened the narrative approach to the concept of personal branding, which “reflects how executives project their identity to others in order to demonstrate their leadership fit” (p. 574). Psychological needs were referenced in five papers, ranging from basic need for self-fulfillment and self-esteem (Shepherd, 2005; Gioia et al., 2014; Zinko and Rubin, 2015) researched by Cohen (1959) and Baumeister and Leary (1995) to non-social motives, as suggested by Labrecque et al. (2011): need for power, to pass time and provide entertainment, and need for advocacy. Finally, Shepherd (2005) and Khedher (2015) suggest that personal branding can be viewed as a self-development tool, grounding their conclusions in Schon's reflective practitioner theory (Schon, 1984).

#### Economic theories

The economic theories, used only in nine papers, help us understand the macro environment, in which personal branding takes place. There are a variety of attempts to describe the current economic conditions shaping social interactions: flexible accumulation (Harvey, 1990), controlled discourse (Andrejevic, 2007), emotional capitalism (Illouz, 2007), leading to the emergence of reputation economy (Gandini, 2016). Hernando and Campo (2017) used Freeman's multi-stakeholder approach to describe the complexity of brand positioning. Spence's signaling theory (Spence, 1973) was used in two papers to reflect communication of unique characteristics to target audiences in imperfect markets.

Thus, we conclude that comprehensive understanding of personal branding lies on four broad social sciences: sociology, marketing, psychology, and economics. Driven by certain needs and shaping own identity (psychological perspective), an individual engages in online and offline interactions with others, trying to manage their perceptions of him/her to gain a certain benefit (sociological perspective). There are specific principles and practices of creating, positioning, and managing own brand (marketing perspective), and these activities are predicated by larger shifts in the organizational and societal contexts (economic perspective).

### Trends, drivers, processes, and outcomes of personal branding

Research on the topic is fragmented, so we used a systematic approach to synthesize the knowledge from the reviewed literature, categorizing the findings into trends, conducive to personal branding, its drivers, related processes, and outcomes. We proceed to discuss these five aspects of personal branding in separate sections below.

#### Trends conducive to personal branding

There are three broad categories of trends that are conducive or preclusive of personal branding activities, found in the reviewed literature: Economic, societal, and technological.

##### Economic (6 papers)

The basic economic premise of an imperfect market (Hernando and Campo, 2017) is already a strong foundation to argue for the need to signal own value to the target audience. Another economic premise for personal branding relates to the economic reality of the modern world. The reviewed literature refers to these conditions as “era of post-Fordism” (Vallas and Cummins, 2015), “knowledge economy” (Gandini, 2016), “sharing economy” (Pera et al., 2016), or “era of consumer-to-consumer” (Chen, 2013), and most concur that the marketplace for skills has become much more demanding, coupled with increasing employment uncertainty (Cederberg, 2017; Holton and Molyneux, 2017) and the rise of portfolio careers (Gandini, 2016), all of which lead to personal branding as an effective career strategy in the new economic environment. Abrate and Viglia (2017) note that “parties operating in sharing economy platforms are incentivized to use reputation-signaling mechanisms to maximize the likelihood of a successful transaction.” (p. 4). Schlosser et al. (2017) conducted their research on career rebranding specifically within the framework of modern career agency, seen as a response to the economic changes. On the other side of the imperfect labor market, employers embrace digital as well, which results in emergence of such practices as, for instance, cybervetting (Berkelaar, 2014). In a similar vein, research by van der Land et al. (2016) shows that effective management of own picture in the LinkedIn profile may lead to better chances of getting a job interview.

##### Societal (4 papers)

Several researchers have attributed the societal shifts to emergence of personal branding. The generational divide and novel lifestyle choices (Harris and Rae, 2011) have contributed to the need of self-promotion, both at work and in private life. Constructing a public image, previously a prerogative of celebrities, today is available to “everyday person” (Eagar and Dann, 2016). Researching social media consumption on YouTube, Chen (2013) maintains that amateur individuals are embracing social media for personal branding purposes. It is noteworthy that different cultures may have varying degrees of appreciation of personal branding practices. For instance, North American blogger communities are more discerning and skeptical of someone's self-promotion activity and they place a greater value on knowledge dissemination, while Middle Eastern personal brander communities are “more praiseworthy, accepting, and less critical of the personal brander efforts at self-promotion and increasing social capital” (Saleem and Iglesias Bedós, 2013, p. 20). Vallas and Christin (2018), having compared the attitudes toward personal branding among the US and French freelance web journalists, report that the French journalists are more wary of such practices than their American counterparts.

##### Technological (6 papers)

There is a widespread consensus that the key driver for personal branding is the ease of access to technology, especially the Web 2.0 tools, such as social media and blogs (Harris and Rae, 2011; Holton and Molyneux, 2017). “If once personal reputation was considered crucial for celebrities and politicians, online tools have allowed personal reputation to become an important marketing task for everyday people” (Pera et al., 2016, p. 45). While technology facilitates personal branding, it also makes it more difficult to differentiate oneself in “hyper-saturated and hyper-fluxed media environment” (Ottovordemgentschenfelde, 2017, p. 65), where digital media skills become an additional kind of brand identity. Green (2016) concurs, having performed research in professional sports area, that, when other “sporting” characteristics are similar, an online profile creates differentiation.

#### Drivers of personal branding

We have identified two broad groups of drivers pertaining to the individual doing own personal branding: Individual and role/industry-related. These factors may explain why, how, and for what reason persons engage in personal branding activities.

##### Individual (5 papers)

Driven by the need for a positive personal reputation (Zinko and Rubin, 2015), comprised of the need for self-esteem, need to belong and desire for rewards, certain personal characteristics, such as attributes and values, make it easier or more difficult for individuals to engage in personal branding. Pihl (2013) performed a netnographic study of three professional Swedish bloggers, which found that individual characteristics aligned with their personal brand enhance its impact and effectiveness. Lorgnier and O'Rourke (2011) identified specific skills required for personal branding: technological, metacognitive, creative and critical. Therefore, we may hypothesize that individuals with superior digital skills, who are able to discover own points of competitive differentiation and creatively turn them into compelling narrative and imagery, while doing that strategically and socially-appropriately, have greater chances of professional and personal success. In addition to that, cultural and social capitals predicate the required effort and the effectiveness of the personal branding process (Khedher, 2015).

##### Role/industry-related (12 papers)

A significant portion of literature links personal branding with the requirements, expectations, and/or limitations of specific roles and industries. Some authors make general statements that professions of today require promoting self via personal branding (Bridgen, 2011; Harris and Rae, 2011), while others discuss specific jobs and industries. We conclude that industries with higher degree of transparency, such as sports (Green, 2016) or journalism (Brems et al., 2016; Holton and Molyneux, 2017), are more conducive to individual personal branding. At the company level, Sturdy and Wright (2008) point out that organizations adopting an enterprise model may be more lenient or even supportive of personal branding. Amoako and Okpattah (2018), having conducted a study on sales executives in the insurance and FMCG sectors in Ghana, suggest that companies investing in personal branding of their employees may gain substantial financial benefits. As the existing research has been focused on particular populations, we observe that those personal branders belong to industries or roles conducive or indifferent to an individual's engaging in personal branding activities. It is logical to assume that some industries or roles, such as defense or police agents, may be less conducive to personal branding or even precluding of such activities. We expose the specific occupations studied, categorized by the degree of conduciveness for personal branding and the type of studied population, in Table 2.

**Table 2:** Samples studied in the reviewed literature, categorized by the degree of conduciveness for personal branding and the type of studied population.

**Conduciveness for personal branding**	**Type of population studied**	**Specific occupations studied**
Highly conducive	Executives, firm owners and high-profile political figures	•CEOs and executives Bendisch et al., 2013; Karaduman, 2013; Fetscherin, 2015; Nolan, 2015; Chen and Chung, 2016; Schlosser et al., 2017; •Entrepreneurs Gandini, 2016; Resnick et al., 2016; Abrate and Viglia, 2017; •Politicians Hughes, 2007; Neale et al., 2008; Omojola, 2008; Balbino et al., 2015; Speed et al., 2015.
	Celebrities	•Writers and artists Tulchinsky, 2011; De la Morena Taboada, 2014; Johns and English, 2016; Hernando and Campo, 2017; •Sportsmen Parmentier and Fischer, 2012; Geurin-Eagleman and Burch, 2016; Green, 2016; Geurin, 2017.
	Academia	•Scholars Noble et al., 2010; Close et al., 2011; Brandabur, 2012; García Montero et al., 2014; Jaring and Bäck, 2017.
Conducive, with possible restrictions	Content-producers	•Journalists Bruns, 2012; Schultz and Sheffer, 2012; Molyneux, 2015; Brems et al., 2016; Hanusch and Bruns, 2017; Hedman, 2017; Holton and Molyneux, 2017; Lopez-Meri and Casero-Ripolles, 2017; Ottovordemgentschenfelde, 2017; Vallas and Christin, 2018; •Bloggers Pihl, 2013; Saleem and Iglesias Bedós, 2013; Delisle and Parmentier, 2016.
	SME owners and self-employed	•Consultants Sturdy and Wright, 2008; Sheikh and Lim, 2011; Pagis and Ailon, 2017; •Psychologists Cederberg, 2017; •Sex workers Phua and Caras, 2008; Cunningham et al., 2018.
Industry and role dependent	Specific roles in organizations	•Librarians Gall, 2012; Baharuddin and Kassim, 2014; •Sales executives and managers Rangarajan et al., 2017; Amoako and Okpattah, 2018.

While a greater number of articles studying executives, firm owners and high-profile political figures was expected, since much management research often begins with the upper echelons, the amount of papers on journalists' personal branding was surprising. We attribute such interest to the fact that journalism of one of the areas most impacted by the advances of social media, with the role and career of journalists currently being in a flux (Holton and Molyneux, 2017). It is worthwhile noting that, according to Brems et al. (2016), freelance journalists are more likely to engage in self-promotion and share personal information than employed journalists. This points to differences in personal branding behaviors even within a specific professional area.

#### Processes of personal branding

Several models are discussed in the reviewed literature regarding the process of personal branding, with a total of 29 papers. Some researchers quote the models from the popular literature, such as Aruda's “extract, express, and exude” (Chen, 2013, p. 334), or the three-step model by McNally and Speak: “(1) identify the areas where your competencies matter; (2) examine your standards and values; (3) define your style” (Gander, 2014, p. 101). Brooks and Anumudu (2016) examined the 10-step model used by the consultancy PriceWaterhouseCoopers to teach personal branding. Other researchers design own approaches such as Resnick et al.'s (2016) “4Ps” self-branding model. Drawing on our analysis of the reviewed papers, we single out the key processes involved in personal branding: raising self-awareness, needs analysis and positioning, constructing brand architecture, self-reflection and feedback-seeking, and sense-making.

##### Raising self-awareness

Self-awareness, introspection and critical skills (Lorgnier and O'Rourke, 2011) are viewed as essential for discovering the “inner self,” a combination of self-identity, personal values and beliefs, self-image, and personal aims (Kucharska, 2017). Self-discovery is the most common first assignment in personal branding courses, discussed in the reviewed literature, and scholars seem to agree that self-awareness is the initial step of the personal branding process (García Montero et al., 2014; Philbrick and Cleveland, 2015; Cederberg, 2017).

##### Needs analysis and positioning

Shepherd (2005) draws our attention to the apparent misalignment between the consumer-oriented approach, advocating for ignoring the “true self” and focusing only on the needs of the target audience, and the personal branding researchers, who advise not to change oneself and build upon individual strengths. He suggests a consensus through engaging in self-reflection vis-à-vis the target audience and the competitors. Two later studies empirically tested applicability of marketing concepts to personal branding in terms of focusing on the target audience and choosing the right positioning strategy. Parmentier et al. (2013) found that to achieve and signal one's capital in the desired organizational field it is necessary to comply with the principles of brand positioning (establishing both points of parity and points of differentiation) and person brand positioning (both fitting into expectations of the field and standing our from competitors in the field). The need for differentiation or uniqueness is highlighted in several papers (Chen, 2013; Gander, 2014; Cederberg, 2017). Such strategies may be specific to various organizational fields and roles. For instance, Parmentier and Fischer (2012) claim that specialization, high-level playing opportunities, revealing publically visible cues about self, and interaction with the audience are key personal branding strategies for professional athletes.

Impression management is the vehicle for positioning the personal brand (Labrecque et al., 2011; Khedher, 2015), which can be achieved through a combination of online and offline strategies. Online activities get the greatest focus from the personal branding scholars, given the changing nature of the economic and social environment and the shift toward digital work; “branding is inevitable when participating in an online environment” (Labrecque et al., 2011, p. 48). Social media and Web 2.0 technology most often discussed in the reviewed literature are Twitter (13 papers), Facebook (6 papers), LinkedIn (5 papers), Instagram (3 papers), blogs (3 papers), and others (5 papers), such as MySpace, About.me, YouTube. As the role of social media in individual career management increases, digital storytelling also comes to the fore as a powerful signaling mechanism of one's worth in the labor market (Jones and Leverenz, 2017).

##### Constructing brand architecture

In studying *professional image*, Roberts (2005) suggested two facets of the construct: *Desired professional image* and *perceived professional image*. We adhere to this line of thinking. A personal brand comprises two key elements: Desired self and perceived identity. *Desired self* can be understood through the dynamic approach to studying work identity (Sveningsson and Alvesson, 2003; Alvesson et al., 2008). While McCall and Simmons (1978) conceptualized *idealized self* as how individuals perceived themselves according to internal values and needs, we posit desired self as how individuals want to be perceived by their target audience. Creating the personal brand is, therefore, similar to what Ibarra and Petriglieri (2010) described as “identity play,” understood as “the crafting and provisional trial of immature (i.e., as yet unelaborated) possible selves” (p. 13).

While most of the papers, discussing personal branding processes, focus on constructing and positioning desired self, only seven articles explicitly address the issue of the audience's perspective, or *perceived identity*, i.e., how in reality one's personal brand is perceived by others (e.g., Cederberg, 2017). In fact, we see this part of personal branding as the most important, as perceptions of others determine their actions toward us.

Gandini (2016) described personal branding as acquisition of reputation, so it is important to understand the concept of personal brand as both what we intend to project to the target audience (desired self), and that audience's reaction to it (perceived identity). Desired self and perceived identity will have all the brand image features, derived from the marketing science: attributes, attitudes, benefits (Keller, 1993), and personality (Aaker, 1997), which Manai and Holmlund (2015) refer to as “brand core,” comprised of core identity (education, skills, personality, values, experience, etc.), extended identity (abilities, attitudes, cultural aspects, etc.) and value proposition (functional, emotional, self-expressive and relationship benefits).

##### Self-reflection and feedback-seeking

These are the two processes that enable the individuals to do maintenance of their personal brands, ensuring their relevance, strength, and competitiveness. Both procure information on the personal brand, the former being internal and the latter—external. Khedher (2015) sees both reflexivity and feedback as integral pieces of the personal branding process. Despite being critical of the way personal branding is being imposed on the society, Wee and Brooks (2010) also see its benefits, as “personal branding strategies are clearly aimed at developing reflexivity because they encourage actors to engage in careful and critical self-assessment about their relative strengths and weaknesses” (p. 47), which is consistent with the research on narrated selfies by Eagar and Dann (2016), confirming that the sheer act of posting a narrated selfie may require a degree of reflexivity. Gioia et al. (2014) states that seeking confirmation on both positive and negative self-conceptions is a natural human behavior, based on the self-verification theory. The nature of the Web 2.0 environment where many personal branding activities take place presupposes a two-way interaction, including receiving feedback (Holton and Molyneux, 2017). Labrecque et al. (2011) considers feedback essential to close the gap between desired self and perceived identity, as it helps avoiding branding failure. Both self-reflection and feedback-seeking lead to greater self-awareness.

##### Sensemaking

As the labor environments become decontextualized, as a consequence of technological advances, people have an increased need to construct their working identities (Brooks and Anumudu, 2016). Cederberg (2017) is more categorical, specifying that “the purpose of a personal brand is to build an identity that associates specific emotions and perceptions with an individual while simultaneously managing these perceptions successfully” (p. 1). People make sense of their environment through their identity (Walsh and Gordon, 2008). Since identity is a collection of meanings attached to a person by self and others (Gecas, 1982), the intelligent career places the onus on the individual to make sense of those meanings. In reality, both individuals and the targets of their personal branding efforts engage in a process of reciprocal sense-making (Gioia et al., 2014).

We posit, therefore, that effective sense-making, feedback-seeking, self-reflection, and greater self-awareness lead to minimizing the gap between desired self and perceived identity, resulting in a stronger and more coherent personal brand.

#### Outcomes of personal branding

While many scholar position personal branding as a career success strategy (Parmentier et al., 2013; Brooks and Anumudu, 2016), the outcomes of personal branding are multifaceted and non-linear. Fifty-one papers specifically identified outcomes of personal branding. Labrecque et al. (2011), acknowledging the importance of career motivation, notes that personal branding can also be used in dating, friendships or merely self-expression. Rangarajan et al. (2017) suggested a list of tangible and intangible measures of the effectiveness of a personal brand in the business setting. We synthesize the outcomes in three categories: individual and organizational, where the individual ones can be either intrinsic or extrinsic. Each category is discussed below. The number in brackets following the name of each category refers to the number of papers that discussed it (we coded “career success” as both intrinsic and extrinsic unless specified).

##### Individual intrinsic outcomes (18 papers)

One of the outcomes of personal branding is developing greater reflexivity (Khedher, 2015). This literature review leads us to conclude that effective personal branding requires self-awareness, feedback-seeking and sense-making, all of which lead to reflexivity in the attempt to position self-identity in the social environment. Some other specifically mentioned intrinsic outcomes are motivation (Ward and Yates, 2013), self-realization (Gandini, 2016), credibility and influence (Ward and Yates, 2013), and acquiring self-promotion skills (Edmiston, 2014). Therefore, we can also hypothesize that effective personal branding leads to greater self-evaluations (self-esteem and general self-efficacy) as defined by Chen et al. (2004).

##### Individual extrinsic outcomes (50 papers)

The majority of the reviewed papers determine the outcomes of personal branding either as furthering professional career (69%, *n* = 22) or creating some sort of social capital (78%, *n* = 25), be it power and influence (Ward and Yates, 2013; Zinko and Rubin, 2015; Hanusch and Bruns, 2017), enhanced visibility (Lee and Cavanaugh, 2016; Jaring and Bäck, 2017), or prestige (Milovanović et al., 2015). Twelve papers identify differentiation as an outcome, which could enable a connection with the target audience (Brems et al., 2016) and use that connection to receive a preferential treatment against those competing for same resources (Parmentier et al., 2013). Ten papers directly point to monetary outcomes of effective personal branding. (Hearn, 2008b) summed up the outcomes of personal branding as, “the function of the branded self is purely rhetorical; its goal is to produce cultural value and, potentially, material profit” (p. 198).

##### Organizational outcomes (10 papers)

Despite the predominant view of personal branding from the position of the benefit for the person, there is emerging research linking employee branding with organizational performance. In a study of 225 Polish professionals, Kucharska and Dąbrowski (2016) found that sharing tacit knowledge, arguably a company's key competitive advantage in the knowledge economy, is positively correlated with personal branding, which is consistent with the exploratory findings of Vosloban (2012). Zinko and Rubin (2015) distill the organizational benefits to three elements: (a) predicting individuals' behaviors, (b) basking in the reflected glory of individuals, and (c) organizational signaling. This applies not only to heads of firms (Chen and Chung, 2016; Malhotra and Malhotra, 2016) or prominent figures in political parties (Neale et al., 2008), but to any employee as personal branding promotes the ideology of enterprise (Sturdy and Wright, 2008).

#### Integration and a conceptual model

**Figure 3:**
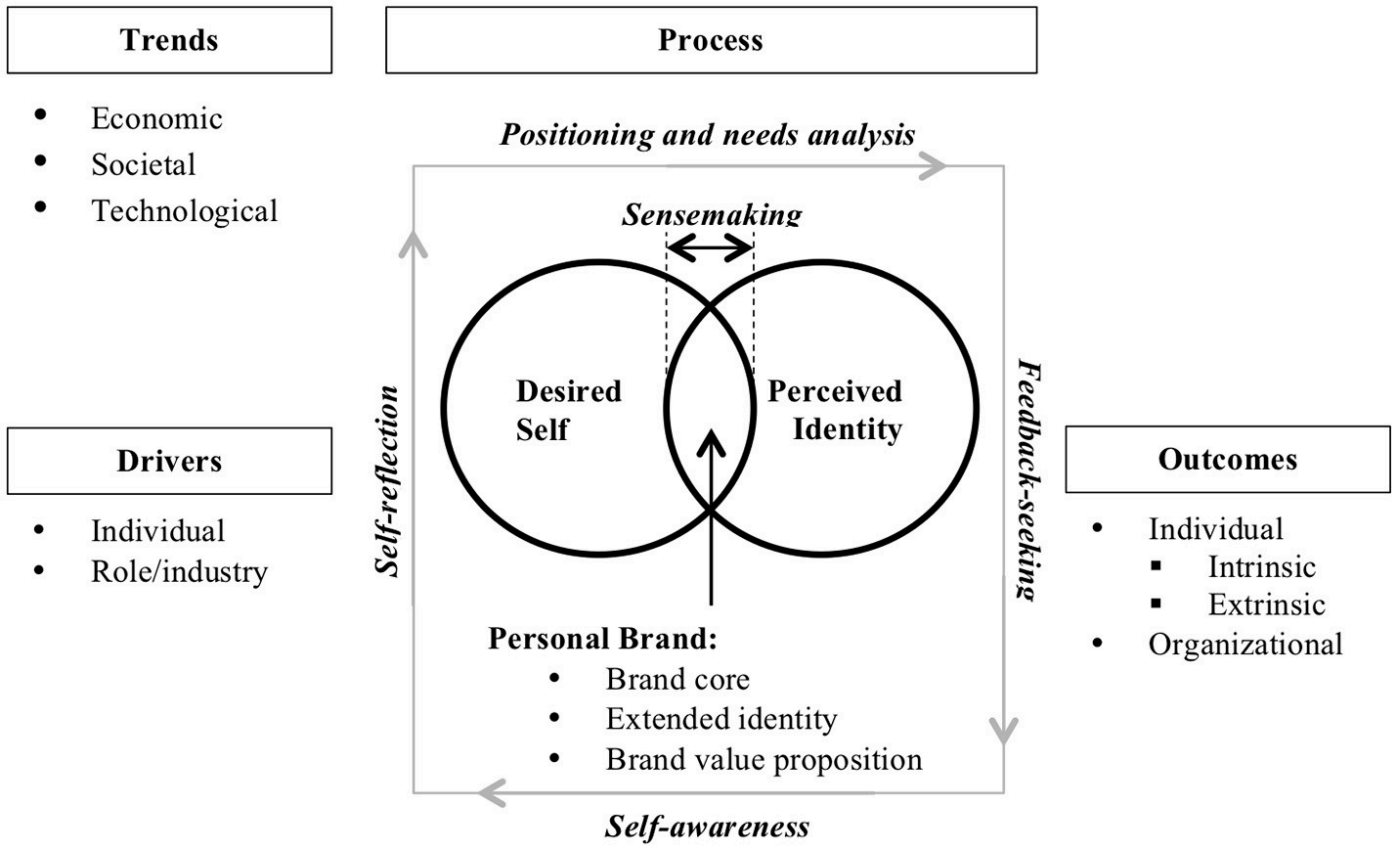
Personal branding model.

Derived from the knowledge in the reviewed literature and the analysis presented above, a conceptual personal branding model emerges as a result. Figure 3 demonstrates the relationships among the key elements of the model, each of which has been discussed above.

By definition, personal branding is a dynamic construct, subject to ongoing adjustment and change. Personal brands need maintenance (Lorgnier and O'Rourke, 2011), i.e., persistent reassessment and monitoring (Cederberg, 2017), which is achieved through constantly repeating the processes described above. This is particularly relevant at the points of career transitions. Schlosser et al. (2017) found that “executives must revisit their personal brands, deciding how to best position their skills and knowledge and values within the context of their new < …> organizations” (p. 576) at each transitional stage. In a study of personal branding in organizational settings Sturdy and Wright (2008) discovered that consultants making a career transition into the corporate labor market need to “trade” their elite personal brand for one that is consistent with the new organization's culture, in order to be effective. When personal branding happens online, the process stages are not discrete and sequential but overarching (Tarnovskaya, 2017), so “when a personal brand is born online, its enforcement and maintenance become critical immediately” (p. 33). All of this evidence leads us to conclude that personal branding is an ongoing process, requiring constant re-evaluation and maintenance.

### Ethical and social considerations

A particular set of findings deals with the ethical and social considerations of personal branding. Irrespective of the definitions, theory, or the model, scholars debate the ethical nature of the branded self in contemporary careers. We have identified four directions of such debate: egalitarianism vs. elitism of personal branding, commodification of self, blurring the line between the personal and professional lives, and teaching personal branding. We proceed to examine these in more detail.

Lair et al. (2005) were the first researchers to raise the ethical questions associated with personal branding, focusing on three areas: gender, race, and culture. They were primarily analyzing at the US labor market, but, e.g., Saleem and Iglesias Bedós (2013) also questioned across the board applicability of personal branding practices in various cultural contents. However, such differences also benefit the individual. Although in a very specific industry of sexual services, Phua and Caras (2008) point out that ethnicity, race, or nationality can be a differentiating factor in personal branding, while gender not being statistically significant. Content analysis of Instagram photos of Olympic athletes revealed that sexually suggestive photos are most popular (Geurin-Eagleman and Burch, 2016). While this lends itself to a discussion on morality of personal branding methods, it also leads us to the conclusion that gender, race and culture issues associated with personal branding are situation-dependent.

The ethical debate of today centers around the concept of commodification with polarized opinions on personal branding as the new *savoir-être* of the new shared, digital and freelance economy (Gandini, 2016) vis-à-vis the self being “a commodity for sale in the labor market, which must generate its own rhetorically persuasive packaging, its own promotional skin, within the confines of the dominant corporate imaginary” (Hearn, 2008b, p. 201). This pervasive messaging to brand oneself may be misused by mass media, e.g., reality television, to take advantage of most “precarious individuals and groups” to expose their insecurities to the public in exchange for creating a stronger personal brand (Hearn, 2008a). Sociologists are concerned that not only our selves become commodified, but also a new type of labor—the digital work of managing own professional identity online—is being thrust on the workers in the realities of post-Fordist capitalism. Vallas and Cummins (2015) even use the word “coercive” to describe the vigor with which personal branding is being introduced to the workforce. They also question the applicability of marketing techniques used for selling shampoo or washing machines to branding individuals. Yet, in their research they found that outward rejection of personal branding was rare, and in general the interviewees demonstrated an “active embrace of branding discourse, coupled with an acknowledgment that one ought to engage in a determined effort to refine one's brand as a condition of one's success and personal fulfillment” (p. 311).

Yet, the requirements of the “knowledge” or “reputation” economy blur the lines between the personal and professional. Labrecque et al. (2011) found that “separating social and professional worlds appears nearly impossible without the proper mechanisms for control” (p. 49). Several studies were conducted around the reporter profession. Conducting interviews with reporters, Molyneux (2015) discovered a sense of uneasiness as they lacked knowledge and skills of balancing professional and personal identities with no clear guidance from their employers. We see that in this specific organizational field, reporters are not aggressively pursuing personal branding, and particularly newspaper reporters being the least motivated to do so (Schultz and Sheffer, 2012). The hypothesis here could be that professions that are most dependent on social media and Web 2.0 technologies require a higher degree of personal branding, while it is less of a necessity for more traditional fields, which is consistent with the research in the entrepreneurial environment (Pihl, 2013; Gandini, 2016). Examining personal brand positioning of journalists on Twitter, Ottovordemgentschenfelde (2017) discovered that they had to manage three identities at the same time—organizational, professional, and personal. This expands the existing role of a worker and adds additional tasks to perform without lowering the employer's performance expectations. This creates a conflict that many employees may not know how to manage. Unfortunately, the popular literature, urging everyone to delve into personal branding, provides little advice on how to deal with such quandaries (Pihl, 2013).

Another ethical point related to the protecting the private space is dissemination of private information. Marwick and boyd (2011) found that social media users operate within the assumption that their imagined audiences are bounded, while, in reality, the cyberspace is limitless. This dialectic pressure between the need to expose oneself in order to self-brand and the need to control own content and the personal boundaries is one of the findings in the study of Labrecque et al. (2011).

Finally, teaching personal branding is a point of concern, too. The issue of the curricula for personal branding and the practical challenge of preparing people to be effective personal branders were raised as early as 2005 in academic sources (Shepherd, 2005). Out of the 100 reviewed articles, 11 deal with teaching personal branding, suggesting various curricula (Edmiston, 2014; Johnson, 2017) and estimating effectiveness of different assignments in teaching personal branding skills (McCorkle and McCorkle, 2012; Wetsch, 2012; Stanton and Stanton, 2013; Jones and Leverenz, 2017). This review demonstrated that there is limited understanding and concurrence on the concepts and processes; therefore teaching unproven ideas raises ethical issues in itself. While some studies report teaching personal branding as a means to developing accompanying skills, such as awareness of online communication issues or metacognitive, creative, and critical thinking skills (Lorgnier and O'Rourke, 2011), most of the papers mentioned in this section teach personal branding as a core subject. For better or worse, the popularity of personal branding has created an industry, which is ahead of the academic thought. Brooks and Anumudu (2016) found that “trainers, career and vocational development consultants, and personal branding enthusiasts publish books and articles and conduct workshops to teach individuals to build their personal brands to become more employable and successful” (p. 24). The contemporary career frameworks (boundaryless, portfolio, intelligent, Protean) share the same underlying assumption that career changes will become more frequent and personal agency will increase. Therefore, such individuals need to be supported by bona fide training on how to thrive in the modern employment environment. The demand has already been vocalized to identify the skills required for effective personal branding (Manai and Holmlund, 2015), develop the content of such training (Lorgnier and O'Rourke, 2011), and provide guidance on the decision to engage in personal branding vs. remaining digitally invisible (Kleppinger and Cain, 2015). However, furthering the ethical debate, Pagis and Ailon (2017) point out that learning the complex personal branding skills may not be accessible to all.

## Discussion and future directions

This systematic review is the first attempt to look at the academic literature pertaining to personal branding comprehensively. Having reviewed the selected 100 papers, we have (a) provided a definition of *personal branding* and a *personal brand* that is more comprehensive, rigorous and detailed than the existing ones and that can help to distinguish these concepts from related ones, and (b) offered a conceptual model capturing inputs, processes, and outputs of personal branding. These findings and this systematic literature review as a whole suggest important directions for future research on personal branding that we discuss below.

### Developing a new measurement instrument of personal branding

While many authors have indicated the need for aligning similar concepts across the related fields (Zinko and Rubin, 2015), as well as developing a comprehensive personal (re-) branding framework (Resnick et al., 2016; Schlosser et al., 2017), only in this paper we have provided an extension to the existing body of research by offering an integrative definition of *personal branding*. By following Podsakoff et al. (2016) rigorous approach toward greater construct clarity through identifying its key attributes and positioning personal branding as a self-standing concept in the nomological field, we outlined its distinct differentiating properties. The introduction of the integrative definition of *personal branding* warrants development of a new measurement instrument of personal branding. While Chen and Chung (2016) already developed a scale to measure the personal brand of a business CEO, we question its validity, due to lack of rigor in the process of scale development and validation. Therefore we hope that the new definition will stimulate much needed personal branding scale development and validation for moving the field further.

### Empirically testing the proposed personal branding model

When developing a conceptual personal branding model, we found that 26 papers discussed the antecedents of personal branding, and 51 papers discussed the outcomes, while only 29 papers focused on the processes. This points toward lacunae in academic knowledge of personal branding that needs further investigation. Understanding the antecedents and outcomes of personal branding is critical for further theory building and field research. By providing an integrative model we offer fresh avenues for future research and join other scholars' calls for empirical testing of conceptual models of personal branding (Bendisch et al., 2013; Dumitriu and Ciobanu, 2015; Johns and English, 2016).

### Studying personal branding in the organizational context

Our review reveals that a small group of researchers specifically point in the direction of studying the person vs. organization tension resulting from personal branding (Hughes, 2007; Bendisch et al., 2013; Karaduman, 2013; Nolan, 2015; Zinko and Rubin, 2015; Ottovordemgentschenfelde, 2017). Only few studies related to the organizational/corporate setting exist (Korzynski, 2012; Vosloban, 2012; Kucharska and Dąbrowski, 2016). Given the discussed tensions between personal and organizational, the managerial attitudes toward employee personal branding call for further research of organizational practices (e.g., guidelines, communication) and employees' activities (e.g., co-branding, signaling). Hence, it may be opportune to converge the studies of careers and human resources management, which traditionally have been apart. Although novel and unconventional, it may prove necessary. Firms must embrace the new reality of workers with strong personal brands overreaching the organizational boundaries. For instance, Kucharska (2017) suggested that the co-branding concept is also applicable to personal brands. So, one of the areas of future research could be examining whether constructing a working identity through personal branding is a source of greater employee loyalty, intrapreneurship intentions, innovation, new clients, and an indication of a stronger employer brand.

### Studying the sustainability and transferability of personal branding

This literature review shows that there is a host of issues regarding the veracity of personal branding (Hughes, 2007), portability of personal brands (Parmentier et al., 2013), and their sustainability (Bendisch et al., 2013). We wish to see further contributions to the ongoing scholarly debate about whether having multiple personal brands is possible, how to adapt one's personal brand when changing employers, and how to avoid the spillover from private social media activities into the professional sphere. Furthermore, up to date the research has only focused on the industries that are most conducive for personal branding. We do not know much about the challenges of creating and maintaining personal brands in settings that are not conducive or outright preclusive of self-promotion, at least, to the outside world. The limited amount of industries and roles studied to date, as well as small samples in those studies, renders scarce opportunities to generalize the knowledge and make conclusive statements about extrapolating the findings. Additionally, the majority of the empirical studies took place in European, Australian, or North American settings, so the possible research directions could lead scholars to test the theoretical premises of personal branding in other cultures.

## Conclusion

We conclude that the academic interest in the concept of personal branding is growing, and that a better understanding of how a personal brand is constructed and managed in the modern labor markets characterized by frequent job changes, project-based work engagements, and increasing job insecurity is needed. This literature review contributes to the field of personal branding by consolidating the extant research, proposing an integrative definition of personal branding and personal brand, developing a conceptual personal branding model, and discussing future research directions that could stimulate the advancement of our knowledge on the topic.

By showing that personal branding is a distinct construct that spans a number of disciplines, we point to an opportunity for a closer integration of traditionally individual-driven career efforts and organization-driven human resources practices to help the employees create effective personal brands, benefitting both the individual and the firm. This paper casts but a glimpse of light into the confusion and uncertainty around the merging spheres of personal and professional. Research and practice have a chance to expand the theory and provide guidance on successfully navigating the current employment reality.

## Author contributions

SG is a PhD candidate, who is the main author of the submitted paper. SK and EL are PhD supervisors. SG was responsible for identifying relevant papers under the supervision of SK, who has expertise in literature review writing. SG also did the initial analysis of the paper and wrote the initial draft. In the consequent process SK and EL helped to develop the paper toward the final submission.

### Conflict of interest statement

The authors declare that the research was conducted in the absence of any commercial or financial relationships that could be construed as a potential conflict of interest.

## References

[B1] Aaker J. L. (1997). Dimensions of brand personality. J. Mark. Res. 34, 347–356. 10.2307/3151897

[B2] Abrate G. Viglia G. (2017). Personal or product reputation? optimizing revenues in the sharing economy. J. Travel Res. 1–13. 10.1177/0047287517741998

[B3] Adams M. (2003). The reflexive self and culture: a critique. Br. J. Sociol. 54, 221–238. 10.1080/0007131032000080212 12945868

[B4] Adams M. (2006). Hybridizing habitus and reflexivity: towards an understanding of contemporary identity? Sociology 40, 511–528. 10.1177/003803850663672

[B5] Alvesson M. Ashcraft K. L. Thomas R. (2008). Identity matters: reflections on the construction of identity scholarship in organization studies. Organization 15, 5–27. 10.1177/1350508407084426

[B6] Amoako G. K. Okpattah B. K. (2018). Unleashing salesforce performance: the impacts of personal branding and technology in an emerging market. Technol. Soc. 54, 20–26. 10.1016/j.techsoc.2018.01.013

[B7] Andrejevic M. (2007). Surveillance in the digital enclosure. Commun. Rev. 10, 295–317. 10.1080/10714420701715365

[B8] Arthur M. B. (2014). The boundaryless career at 20: where do we stand, and where can we go? Career Dev. Int. 19, 627–640. 10.1108/CDI-05-2014-0068

[B9] Arthur M. B. Khapova S. N. Richardson J. (2017). An Intelligent Career: Taking Ownership of Your Work and Your Life. New York, NY: Oxford University Press.

[B10] Arthur M. B. Khapova S. N. Wilderom C. P. M. (2005). Career success in a boundaryless career world. J. Organ. Behav. 26, 177–202. 10.1002/job.290

[B11] Arthur M. B. Rousseau D. M. (1996). The Boundaryless Career. New York, NY: Oxford University Press.

[B12] Baharuddin M. F. Kassim N. A. (2014). Conceptualizing personal branding for librarians, Paper Presented at the 23rd International-Business-Information-Management-Association Conference on Visio 2020: Sustainable Growth, Economic Development, and Global Competitiveness (Valencia). Available online at: https://www.scopus.com/record/display.uri?eid=2-s2.0-84906264309andorigin=inwardandtxGid=f485a98005c5cbbc865577f1dda3859d

[B13] Balbino T. Neto J. de Aquino A. P. P. (2015). Personal Style: a strategic tool for public relations. Rev. Int. Relac. Pub. 5, 207–228. 10.5783/rirp-9-2015-11-207-228

[B14] Baumeister R. F. (1982). A self-presentational view of social phenomena. Psychol. Bull. 91:3 10.1037/0033-2909.91.1.3

[B15] Baumeister R. F. Leary M. R. (1995). The need to belong: desire for interpersonal attachments as a fundamental human motivation. Psychol. Bull. 117:497 10.1037/0033-2909.117.3.497 7777651

[B16] Beck U. (1992). Risk Society: Towards a New Modernity, Vol. 17. London: Sage.

[B17] Bendisch F. Larsen G. Trueman M. (2013). Fame and fortune: a conceptual model of CEO brands. Eur. J. Mark. 47, 596–614. 10.1108/03090561311297472

[B18] Berkelaar B. L. (2014). Cybervetting, online information, and personnel selection. Manage. Commun. Q. 28, 479–506. 10.1177/0893318914541966

[B19] Bolino M. Long D. Turnley W. (2016). Impression management in organizations: critical questions, answers, and areas for future research. Annu. Rev. Organ. Psychol. Organ. Behav. 3, 377–406. 10.1146/annurev-orgpsych-041015-062337

[B20] Bourdieu P. (1993). The Field of Cultural Production: Essays on Art and Literature. New York, NY: Columbia University Press.

[B21] Brandabur R. E. (2012). Personal branding of a teacher - an approach into e-educational environment, in Paper Presented at the 8th International Scientific Conference eLearning and Software for Education (Bucharest).

[B22] Brems C. Temmerman M. Graham T. Broersma M. (2016). Personal branding on Twitter. Digit. J. 5, 443–459. 10.1080/21670811.2016.1176534

[B23] Bridgen L. (2011). Emotional labour and the pursuit of the personal brand: public relations practitioners' use of social media. J. Media Pract. 12, 61–76. 10.1386/jmpr.12.1.61_1

[B24] Brooks A. K. Anumudu C. (2016). Identity development in personal branding instruction. Adult Learn. 27, 23–29. 10.1177/1045159515616968

[B25] Bruns A. (2012). Journalists and Twitter: how australian news organisations adapt to a new medium. Media Int. Austr. 144, 97–107. 10.1177/1329878X1214400114

[B26] Cawsey T. F. (1995). The portfolio career as a response to a changing job market. J. Career Plan. Employ. 56, 41–46.

[B27] Cederberg C. D. (2017). Personal branding for psychologists: ethically navigating an emerging vocational trend. Prof. Psychol. Res. Pract. 48, 183–190. 10.1037/pro0000129

[B28] Chen C.-P. (2013). Exploring personal branding on YouTube. J. Internet Comm. 12, 332–347. 10.1080/15332861.2013.859041

[B29] Chen G. Gully S. M. Eden D. (2004). General self-efficacy and self-esteem: toward theoretical and empirical distinction between correlated self-evaluations. J. Organ. Behav. 25, 375–395. 10.1002/job.251

[B30] Chen H. M. Chung H. M. (2016). How to measure personal brand of a business CEO. J. Hum. Resour. Sustain. Stud. 4, 305–324. 10.4236/jhrss.2016.44030

[B31] Close A. G. Moulard J. G. Monroe K. B. (2011). Establishing human brands: determinants of placement success for first faculty positions in marketing. Acad. Mark. Sci. J. 39, 922–941. 10.1007/s11747-010-0221-6

[B32] Cohen A. R. (1959). Some implications of self-esteem for social influence, in Personality and Persuasibility, eds Hovland C. I. Janis I. L. (Oxford: Yale Univer. Press), 102–120.

[B33] Cunningham S. Sanders T. Scoular J. Campbell R. Pitcher J. Hill K.. (2018). Behind the screen: commercial sex, digital spaces and working online. Technol. Soc. 53, 47–54. 10.1016/j.techsoc.2017.11.004

[B34] De la Morena Taboada M. (2014). Evolución del concepto de marca personal. Análisis de la repercusión de la prensa en la creación de marca personal en la época victoriana. Hist. Comun. Soc. 19, 393–401. 10.5209/rev_HICS.2014.v19.44965

[B35] Delisle M.-P. Parmentier M.-A. (2016). Navigating person-branding in the fashion blogosphere. J. Glob. Fashion Mark. 7, 211–224. 10.1080/20932685.2016.1167619

[B36] Du Gay P. (1996). Consumption and Identity at Work. London: Sage.

[B37] Dumitriu D.-L. Ciobanu C. V. (2015). Personal branding: the marketization of self in the digital landscape, in Strategica: Local versus Global, eds Brǎtianu C. Zbuchea A. Pînzaru F. Vǎtǎmǎnescu E.-M. Leon R.-D. (Bucharest), 686–693.

[B38] Eagar T. Dann S. (2016). Classifying the narrated #selfie: genre typing human-branding activity. Eur. J. Mark. 50, 1835–1857. 10.1108/EJM-07-2015-0509

[B39] Edmiston D. (2014). Creating a personal competitive advantage by developing a professional online presence. Mark. Educ. Rev. 24, 21–24. 10.2753/MER1052-8008240103

[B40] Erikson E. H. (1968). Identity: Youth and Crisis. New York, NY: WW Norton and Company.

[B41] Evans J. R. (2017). A strategic approach to self-branding. J. Glob. Schol. Mark. Sci. 27, 270–311. 10.1080/21639159.2017.1360146

[B42] Fetscherin M. (2015). The CEO branding mix. J. Bus. Strat. 36, 22–28. 10.1108/JBS-01-2015-0004

[B43] Gall D. (2012). Librarian like a rock star: using your personal brand to promote your services and reach distant users. J. Libr. Adm. 52, 549–558. 10.1080/01930826.2012.707952

[B44] Gander M. (2014). Managing your personal brand. Perspectives 18, 99–102. 10.1080/13603108.2014.913538

[B45] Gandini A. (2016). Digital work: self-branding and social capital in the freelance knowledge economy. Mark. Theory 16, 123 10.1177/1470593115607942

[B46] García Montero E. De la Morena Taboada M. Presol Herrero Á. (2014). Aplicación del autoconcepto al desarrollo de la marca personal. Análisis comparativo entre estudiantes internacionales. Hist. Comun. Soc. 19, 819–833. 10.5209/rev_HICS.2014.v19.46561

[B47] Gardner W. L. Martinko M. J. (1988). Impression management in organizations. J. Manage. 14, 321–338. 10.1177/014920638801400210

[B48] Gay P. D. Salaman G. (1992). The cult[ure] of the customer. J. Manage. Stud. 29, 615–633. 10.1111/j.1467-6486.1992.tb00681.x

[B49] Gecas V. (1982). The self-concept. Annu. Rev. Sociol. 8, 1–33. 10.1146/annurev.so.08.080182.000245

[B50] Geurin A. N. (2017). Elite female athletes' perceptions of new media use relating to their careers: a qualitative analysis. J. Sport Manage. 31, 345–359. 10.1123/jsm.2016-0157

[B51] Geurin-Eagleman A. N. Burch L. M. (2016). Communicating via photographs: a gendered analysis of Olympic athletes' visual self-presentation on Instagram. Sport Manage. Rev. 19, 133–145. 10.1016/j.smr.2015.03.002

[B52] Giddens A. (1991). Modernity and Self-Identity: Self and Society in the Late Modern Age. Stanford, CA: Stanford University Press.

[B53] Gioia D. A. Hamilton A. L. Patvardhan S. D. (2014). Image is everything. Res. Organ. Behav. 34, 129–154. 10.1016/j.riob.2014.01.001

[B54] Goffman E. (1959). The Presentation of Self in Everyday Life. 1st Edn New York, NY: Anchor Books.

[B55] Green M. R. (2016). The impact of social networks in the development of a personal sports brand. Sport Bus. Manage. 6, 274–294. 10.1108/SBM-09-2015-0032

[B56] Greenhalgh T. Peacock R. (2005). Effectiveness and efficiency of search methods in systematic reviews of complex evidence: audit of primary sources. BMJ 331, 1064–1065. 10.1136/bmj.38636.593461.68 16230312 PMC1283190

[B57] Greenhaus J. H. Kossek E. E. (2014). The contemporary career: a work–home perspective. Annu. Rev. Organ. Psychol. Organ. Behav. 1, 361–388. 10.1146/annurev-orgpsych-031413-091324

[B58] Hanusch F. Bruns A. (2017). Journalistic branding on twitter: a representative study of Australian journalists' profile descriptions. Digi. J. 5, 26–43. 10.1080/21670811.2016.1152161

[B59] Harris L. Rae A. (2011). Building a personal brand through social networking. J. Bus. Strat. 32, 14–21. 10.1108/02756661111165435

[B60] Harvey D. (1990). Flexible accumulation through urbanization reflections on“ post-modernism” in the american city. Perspecta 251–272. 10.2307/1567167

[B61] Hearn A. (2008a). Insecure: narratives and economies of the branded self in transformation television. Continuum J. Media Cult. Stud. 22, 495–504. 10.1080/10304310802189972

[B62] Hearn A. (2008b). Meat, mask, burden. J. Consum. Cult. 8, 197–217. 10.1177/1469540508090086

[B63] Hedman U. (2017). Making the most of Twitter How technological affordances influence Swedish journalists' self-branding. Journalism Theory Practi. Crit. 2, 1–18. 10.1177/1464884917734054

[B64] Hernando E. Campo S. (2017). Does the artist's name influence the perceived value of an art work? Int. J. Arts Manage. 19, 46–58.

[B65] Hodge C. Walker M. (2015). Personal branding: a perspective from the professional athlete-level-of-analysis. Int. J. Sport Manage. Mark. 16, 112–131. 10.1504/IJSMM.2015.074920

[B66] Holton A. E. Molyneux L. (2017). Identity lost? The personal impact of brand journalism. Journalism 18, 195–210. 10.1177/1464884915608816

[B67] Hughes A. (2007). Personal brands: an exploratory analysis of personal brands in Australian political marketing, in Paper Presented at the Australia and New Zealand Marketing Academy Conference 2007 (Dunedin).

[B68] Ibarra H. (1999). Provisional selves: experimenting with image and identity in professional adaptation. Adm. Sci. Q. 44, 764–791. 10.2307/2667055

[B69] Ibarra H. Petriglieri J. L. (2010). Identity work and play. J. Organ. Change Manage. 23, 10–25. 10.1108/09534811011017180

[B70] Illouz E. (2007). Cold Intimacies: The Making of Emotional Capitalism. Cambridge: Polity Press.

[B71] Jaring P. Bäck A. (2017). How researchers use social media to promote their research and network with industry. Technol. Innov. Manage. Rev. 7, 32–39. 10.22215/timreview/1098

[B72] Johns R. English R. (2016). Transition of self: repositioning the celebrity brand through social media—the case of Elizabeth Gilbert. J. Bus. Res. 69, 65–72. 10.1016/j.jbusres.2015.07.021

[B73] Johnson K. M. (2017). The importance of personal branding in social media: educating students to create and manage their personal brand. Int. J. Educ. Soc. Sci. 4, 21–27.

[B74] Jones B. Leverenz C. (2017). Building personal brands with digital storytelling eportfolios. Int. J. ePortfolio 7, 67–91.

[B75] Karaduman I. (2013). The effect of social media on personal branding efforts of top level executives. Proc. Soc. Behav. Sci. 99, 465–473. 10.1016/j.sbspro.2013.10.515

[B76] Keller K. L. (1993). Conceptualizing, measuring, and managing customer-based brand equity. J. Market. 57, 1–22. 10.2307/1252054

[B77] Khedher M. (2015). A brand for everyone: guidelines for personal brand managing. J. Glob. Bus. Issues 9, 19–27.

[B78] Kleppinger C. A. Cain J. (2015). Personal digital branding as a professional asset in the digital age. Am. J. Pharm. Educ. 79, 79–79. 10.5688/ajpe79679 26430266 PMC4584371

[B79] Korzynski P. (2012). Leading people and leading authentic self through online networking platforms. Actual Probl. Econ. 133, 231–241.

[B80] Kowalski R. M. Leary M. R. (1990). Strategic self-presentation and the avoidance of aversive events: antecedents and consequences of self-enhancement and self-depreciation. J. Exp. Soc. Psychol. 26, 322–336. 10.1016/0022-1031(90)90042-K

[B81] Kucharska W. (2017). Consumer social network brand identification and personal branding. How do social network users choose among brand sites? Cogent. Bus. Manage. 4, 1–19. 10.1080/23311975.2017.1315879

[B82] Kucharska W. Dąbrowski J. (2016). Tacit knowledge sharing and personal branding: how to derive innovation from project teams?, in 11th European Conference on Innovation and Entrepreneurship (Reading: Academic Conferences and Publishing International Limited). 10.13140/RG.2.2.25473.86885

[B83] Labrecque L. I. Markos E. Milne G. R. (2011). Online personal branding: processes, challenges, and implications. J. Interact. Mark. 25, 37–50. 10.1016/j.intmar.2010.09.002

[B84] Lair D. J. Sullivan K. Cheney G. (2005). Marketization and the recasting of the professional self: the rhetoric and ethics of personal branding. Manage. Commun. Q. 18, 307–343. 10.1177/0893318904270744

[B85] Lee J. W. Cavanaugh T. (2016). Building your brand: the integration of infographic resume as student self-analysis tools and self-branding resources. J. Hosp. Leisure Sport Tourism Educ. 18, 61–68. 10.1016/j.jhlste.2016.03.001

[B86] Lopez-Meri A. Casero-Ripolles A. (2017). Journalists' strategies to build personal brand on Twitter: positioning, content curation, personalization and specialisation. Rev. Mediter. Comun. J. Commun. 8, 59–73. 10.14198/MEDCOM2017.8.1.5

[B87] Lorgnier N. O'Rourke S. (2011). Improving students communication skills and awareness online, an opportunity to enhance learning and help personal branding, in Paper Presented at the 5th International Technology, Education and Development Conference (Valencia).

[B88] Malhotra C. K. Malhotra A. (2016). How CEOs can leverage Twitter. MIT Sloan Manage. Rev. 57, 73–79.

[B89] Manai A. Holmlund M. (2015). Self-marketing brand skills for business students. Mark. Intell. Plan. 33, 749–762. 10.1108/MIP-09-2013-0141

[B90] Marwick A. boyd d. (2011). I tweet honestly, I tweet passionately: Twitter users, context collapse, and the imagined audience. New Media Soc. 13, 114–133. 10.1177/1461444810365313

[B91] McCall G. J. Simmons J. L. (1978). Identities and Interactions. New York, NY: Free Press.

[B92] McCorkle D. E. McCorkle Y. L. (2012). Using linkedin in the marketing classroom: exploratory insights and recommendations for teaching social media/networking. Mark. Educ. Rev. 22, 157–166. 10.2753/MER1052-8008220205

[B93] Mead G. H. (1934). Mind, self and society, Vol. 111: Chicago: University of Chicago Press.

[B94] Meyrowitz J. (1990). Redefining the situation: extending dramaturgy into a theory of social change and media effects, in Beyond Goffman: Studies on Communication, Institution, and Social Interaction, ed Riggins S. H. (Berlin: Mouton de Gruyter), 65–97.

[B95] Miles S. J. Mangold G. (2004). A conceptualization of the employee branding process. J. Relat. Mark. 3, 65–87. 10.1300/J366v03n02_05

[B96] Milovanović S. Baltazarević B. Milovanović N. (2015). Personal branding through leadership. Int. Rev. 3–4, 75–81. 10.5937/intrev1504075M

[B97] Moher D. Liberati A. Tetzlaff J. Altman D. G. (2009). Preferred reporting items for systematic reviews and meta-analyses: the PRISMA statement. Ann. Intern. Med. 151, 264. 10.7326/0003-4819-151-4-200908180-00135 19622511

[B98] Mol E. Khapova S. N. Elfring T. (2015). Entrepreneurial team cognition: a review. Int. J. Manage. Rev. 17, 232–255. 10.1111/ijmr.12055

[B99] Molyneux L. (2015). What journalists retweet: opinion, humor, and brand development on Twitter. Journalism 16, 920–935. 10.1177/1464884914550135

[B100] Montoya P. Vandehey T. (2002). The Brand Called You. Nightingale Conant.

[B101] Neale L. Hughes A. Dann S. M. (2008). Exploring the application of personal brands and opinion leadership in political marketing, in Paper Presented at the ANZMAC 2008, University of Western Sydney (Sydney), Available online at: http://eprints.qut.edu.au

[B102] Noble C. H. Bentley J. P. Campbell D. Singh J. J. (2010). In search of eminence: a personal brand-building perspective on the achievement of scholarly prominence in marketing. J. Mark. Educ. 32, 314–327. 10.1177/0273475310379337

[B103] Nolan L. (2015). The impact of executive personal branding on non-profit perception and communications. Public Relat. Rev. 41, 288–292. 10.1016/j.pubrev.2014.11.001

[B104] Omojola O. (2008). Audience mindset and influence on personal political branding. J. Soc. Sci. 16, 127–134. 10.1080/09718923.2008.11892609

[B105] Ottovordemgentschenfelde S. (2017). 'Organizational, professional, personal': an exploratory study of political journalists and their hybrid brand on Twitter. Journalism 18, 64–80. 10.1177/1464884916657524

[B106] Pagis M. Ailon G. (2017). The paradoxes of self-branding. Work Occup. 44, 243–267. 10.1177/0730888417709327

[B107] Parmentier M.-A. s Fischer E. Reuber A. R. (2013). Positioning person brands in established organizational fields. J. Acad. Mark. Sci. 41, 373–387. 10.1007/s11747-012-0309-2

[B108] Parmentier M.-A. S. Fischer E. (2012). How athletes build their brands. Int. J. Sport Manage. Marketi. 11, 106–124. 10.1504/IJSMM.2012.045491

[B109] Pera R. Viglia G. Furlan R. (2016). Who am I? How compelling self-storytelling builds digital personal reputation. J. Interact. Mark. 35, 44–55. 10.1016/j.intmar.2015.11.002

[B110] Peters T. (1997). The brand called you. Fast Company 10, 83–90.

[B111] Philbrick J. L. Cleveland A. D. (2015). Personal branding: building your pathway to professional success. Med. Ref. Serv. Q. 34, 181–189. 10.1080/02763869.2015.1019324 25927510

[B112] Phua V. C. Caras A. (2008). Personal brand in online advertisements: comparing white and brazilian male sex workers. Sociol. Focus 41, 238–255. 10.1080/00380237.2008.10571333

[B113] Pihl C. (2013). In the borderland between personal and corporate brands - the case of professional bloggers. J. Glob. Fashion Mark. 4, 112–127. 10.1080/20932685.2013.763474

[B114] Podsakoff P. M. MacKenzie S. B. Podsakoff N. P. (2016). Recommendations for creating better concept definitions in the organizational, behavioral, and social sciences. Organ. Res. Methods 19, 159–203. 10.1177/1094428115624965

[B115] Rampersad H. K. (2008). A new blueprint for powerful and authentic personal branding. Perform. Improv. 47, 34–37. 10.1002/pfi.20007

[B116] Rangarajan D. Gelb B. D. Vandaveer A. (2017). Strategic personal branding—and how it pays off. Bus. Horiz. 60, 657–666. 10.1016/j.bushor.2017.05.009

[B117] Resnick S. M. Cheng R. Simpson M. Lourenço F. (2016). Marketing in SMEs: a “4Ps” self-branding model. Int. J. Entrepren. Behav. Res. 22, 155–174. 10.1108/ijebr-07-2014-0139

[B118] Roberts L. M. (2005). Changing faces: Professional image construction in diverse organizational settings. Acad. Manage. Rev. 30, 685–711. 10.5465/amr.2005.18378873

[B119] Saleem F. Iglesias Bedós O. (2013). Online personal branding in the Middle East and North America: a comparison of social capital accumulation and community response, in Paper Presented at the 2013 AMS Annual Conference (Monterey, CA).

[B120] Schlenker B. R. (1980). Impression Management. Monterey, CA: Brooks/Cole Publishing Company.

[B121] Schlosser F. McPhee D. M. Forsyth J. (2017). Chance events and executive career rebranding: implications for career coaches and nonprofit HRM. Hum. Resour. Manage. 56, 571–591. 10.1002/hrm.21789

[B122] Schon D. A. (1984). The Reflective Practitioner: How Professionals Think in Action, Vol. 5126. New York, NY: Basic Books.

[B123] Schultz B. Sheffer M. L. (2012). Personal branding still in future for most newspaper reporters. Newsp. Res. J. 33, 63–77. 10.1177/073953291203300406

[B124] Sheikh A. Lim M. (2011). Engineering consultants' perceptions of corporate branding: A case study of an international engineering consultancy. Indus. Mark. Manage. 40, 1123–1132. 10.1016/j.indmarman.2011.09.006

[B125] Shepherd I. D. H. (2005). From cattle and coke to charlie: meeting the challenge of self marketing and personal branding. J. Mark. Manage. 21, 589–606. 10.1362/0267257054307381

[B126] Speed R. Butler P. Collins N. (2015). Human branding in political marketing: applying contemporary branding thought to political parties and their leaders. J. Polit. Market. 14, 129–151. 10.1080/15377857.2014.990833

[B127] Spence M. (1973). Job market signaling. Q. J. Econ. 87, 355–374. 10.2307/1882010

[B128] Stanton A. D. A. Stanton W. W. (2013). Building “Brand Me”: creating a personal brand statement. Mark. Educ. Rev. 23, 81–85. 10.2753/MER1052-8008230113

[B129] Sturdy A. Wright C. (2008). A consulting diaspora? Enterprising selves as agents of enterprise. Organization 15, 427–444. 10.1177/1350508408088538

[B130] Suddaby R. (2010). Editor's comments: construct clarity in theories of management and organization. Acad. Manage. Rev. 35, 346–357. 10.5465/amr.35.3.zok346

[B131] Sveningsson S. Alvesson M. (2003). Managing managerial identities: organizational fragmentation, discourse and identity struggle. Hum. Relat. 56, 1163–1193. 10.1177/00187267035610001

[B132] Tarnovskaya V. (2017). Reinventing personal branding building a personal brand through content on YouTube. J. Int. Bus. Res. Mark. 3, 29–35. 10.18775/jibrm.1849-8558.2015.31.3005

[B133] Thomson M. (2006). Human brands: Investigating antecedents to consumers' strong attachments to celebrities. J. Mark. 70, 104–119. 10.1509/jmkg.70.3.104

[B134] Tulchinsky G. (2011). Fantasy and personal branding: market dynamics and stylistic integration of the popular literature. J. Sociol. Soc. Anthropol. XIV, 364–372.

[B135] Turner J. C. Oakes P. J. (1986). The significance of the social identity concept for social psychology with reference to individualism, interactionism and social influence. Br. J. Soc. Psychol. 25, 237–252. 10.1111/j.2044-8309.1986.tb00732.x

[B136] Vallas S. P. Christin A. (2018). Work and identity in an era of precarious employment: how workers respond to “personal branding” discourse. Work Occup. 45, 3–37. 10.1177/0730888417735662

[B137] Vallas S. P. Cummins E. R. (2015). Personal branding and identity norms in the popular business press: enterprise culture in an age of precarity. Organ. Stud. 36, 293–319. 10.1177/0170840614563741

[B138] van der Land S. F. Willemsen L. M. Wilton B. G. E. (2016). Professional personal branding, in HCI in Business, Government, and Organizations: eCommerce and Innovation. HCIBGO 2016. Lecture Notes in Computer Science, Vol 9751, eds Nah F. H. Tan C. H. (Cham: Springer), 10.1007/978-3-319-39396-4_11

[B139] Vitberg A. (2010). Developing your personal brand equity. J. Account. 210, 42–45, 48.

[B140] Vosloban R. I. (2012). Employee's personal branding as a competitive advantage – a managerial approach. Int. J. Manage. Sci. Inform. Technol. II, 147–159.

[B141] Walsh K. Gordon J. R. (2008). Creating an individual work identity. Hum. Resourc. Manage. Rev. 18, 46–61. 10.1016/j.hrmr.2007.09.001

[B142] Ward C. Yates D. (2013). Personal branding and e-professionalism. J. Serv. Sci. 6, 101–104. 10.19030/jss.v6i1.8240

[B143] Wee L. Brooks A. (2010). Personal branding and the commodification of reflexivity. Cult. Sociol. 4, 45–62. 10.1177/1749975509356754

[B144] Wetsch L. R. (2012). A personal branding assignment using social media. J. Advert. Educ. 16, 30–36. 10.1177/109804821201600106

[B145] Zinko R. Rubin M. (2015). Personal reputation and the organization. J. Manage. Organ. 21, 217–236. 10.1017/jmo.2014.76

