# Supplementary material for: Personal Branding: Interdisciplinary Systematic Review and Research Agenda
Source: Front Psychol. 2018 Nov 21;9:2238. doi: 10.3389/fpsyg.2018.02238 (PMC6258780; doi:10.3389/fpsyg.2018.02238)
Supplement: Supplementary file 1 [file Table_1.docx]

Supplementary Material

Personal branding: Systematic review and extension

Sergey Gorbatov^1*^, Svetlana N. Khapova^1^, Evgenia I. Lysova^1^

^1^ Vrije Universiteit Amsterdam, De Boelelaan 1105, 1085HV, Amsterdam, the Netherlands

*** Correspondence:**Sergey Gorbatov
sergey.gorbatov@student.vu.nl

**Supplementary Table 1.** *Papers included in the review. In the Definition column, <PBing> refers to Personal Branding and <PB> to Personal Brand.*

| Author | Year | Journal | Title | Definition | Nature | Population studied |
| --- | --- | --- | --- | --- | --- | --- |
| Lair et al. | 2005 | Management Communication Quarterly | Marketization and the Recasting of the Professional Self: The Rhetoric and Ethics of Personal Branding | <PBing> programmatic approach to the selling of product, service, organization, cause, or person that is fashioned as a proactive response to the emerging desires of a target audience or market | Conceptual | N/A |
| Shepherd | 2005 | Journal of Marketing Management | From cattle and Coke to Charlie: meeting the challenge of self marketing and personal branding. | <PBing> varied activities undertaken by individuals to make themselves known in the marketplace, usually for the purpose of obtaining gainful employment | Conceptual | N/A |
| Hughes | 2007 | ANZMAC | Personal Brands: An Exploratory Analysis of Personal Brands in Australian Political Marketing | <PB> a person, name, term, sign, symbol or design, or a combination of these, intended to identify the goods or services of one seller, or group of sellers, and to differentiate them from those of competitors | Conceptual | N/A |
| Phua & Caras | 2008 | Sociological Focus | Personal Brand in Online Advertisements: Comparing White and Brazilian Male Sex Workers | Same as Shepherd (2005) | Qualitative | Sex workers |
| Omojola | 2008 | Journal of Social Sciences | Audience Mindset and Influence on Personal Political Branding | <PBing>creation and development of distinct values <…> in a manner that makes it appealing and different from others, with an objective of giving it an easily recognized clear identity, <...> communicated to the target customers also in a manner that elicits goodwill with them <PB> the sum total of proprietary visual, emotional, natural and cultural image or attributes associated with a person, company, product or service | Qualitative | Politicians |
| Neale et al. | 2008 | ANZMAC | Exploring the application of personal brands and opinion leadership in political marketing | Same as Hughes (2007) | Conceptual | Politicians |
| Hearn | 2008 | Journal Of Consumer Culture | `Meat, Mask, Burden` | <PBing> creating a detachable, saleable image or narrative, which effectively circulates cultural meanings <PB> a rhetorically persuasive version of yourself; a form of self-presentation singularly focused on attracting attention and acquiring cultural and monetary value | Conceptual | N/A |
| Hearn | 2008 | Journal of Media & Cultural Studies | Insecure: Narratives and economies of the branded self in transformation television. | <PBing> a distinct kind of labor, involving an outer-directed process of highly stylized self-construction | Conceptual | N/A |
| Rampersad | 2008 | Performance Improvement | A New Blueprint for Powerful and Authentic Personal Branding | <PB> synthesis of all expectations, images, and perceptions it creates in the minds of others when they see or hear your name. | Conceptual | N/A |
| Sturdy & Wright | 2008 | Organization | A consulting diaspora? Enterprising selves as agents of enterprise | N/A | Qualitative | Consultants |
| Wee & Brooks | 2010 | Cultural Sociology | Personal Branding and the Commodification of Reflexivity | <PB> impression that others may have of him/her | Conceptual | N/A |
| Noble et al. | 2010 | Journal of Marketing Education | In Search of Eminence: A Personal Brand-Building Perspective on the Achievement of Scholarly Prominence in Marketing | <PBing> building a reputation of eminence for oneself | Quantitative | Academia (scholars) |
| Harris & Rae | 2011 | Journal Of Business Strategy | Building A Personal Brand Through Social Networking | <PB> a mix of reputation, trust, attention and execution | Conceptual | N/A |
| Labrecque et al. | 2011 | Journal Of Interactive Marketing | Online Personal Branding: Processes, Challenges, And Implications | <PBing> capturing and promoting an individual's strengths and uniqueness to a target audience | Mixed methods | General public |
| Marwick & Boyd | 2011 | New Media & Society | I tweet honestly, I tweet passionately: Twitter users, context collapse, and the imagined audience | <PBing> strategically appealing to followers becomes a carefully calculated way to market oneself as a commodity in response to employment uncertainty | Qualitative | General public |
| Sheikh & Lim | 2011 | Industrial Marketing Management | Engineering Consultants' Perceptions Of Corporate Branding: A Case Study Of An International Engineering Consultancy | N/A | Qualitative | Consultants |
| Tulchinsky | 2011 | The Journal of Sociology and Social Anthropology | Fantasy and Personal Branding: Market Dynamics and Stylistic Integration of the Popular Literature | <PB> the promise to bring about the desired experiences | Conceptual | Celebrities |
| Lorgnier et al. | 2011 | INTED2011 | Improving Students' Communication Skills And Awareness Online: An Opportunity To Enhance Learning And Help Personal Branding | <PBing> process by which individuals and entrepreneurs differentiate themselves and stand out from the crowd by identifying and articulating their unique value proposition | Qualitative | Academia (students) |
| Close et al. | 2011 | Journal Of The Academy Of Marketing Science | Establishing Human Brands: Determinants Of Placement Success For First Faculty Positions In Marketing | <Human brand> persona, well-known or emerging, who are the subject of marketing, interpersonal or inter-organizational communications | Quantitative | Academia (students) |
| Parmentier & Fisher | 2012 | International Journal Of Sport Management And Marketing | How athletes build their brands | <PB> the set of associations identified with a particular person | Qualitative | Sportsmen |
| Vosloban et al. | 2012 | Business Excellence Challenges During The Economic Crisis | Employee'S Personal Branding As A Competitive Advantage - A Managerial Approach | N/A | Qualitative | Managers |
| Korzynski | 2012 | Actual Problems Of Economics | Leading People And Leading Authentic Self Through Online Networking Platforms | N/A | Quantitative | Professionals |
| Gall | 2012 | Journal of Library Administration | Librarian Like a Rock Star: Using Your Personal Brand to Promote Your Services and Reach Distant Users | <PB> an idea in the mind of your constituents created by what you say and do | Conceptual | Librarians |
| Bruns | 2012 | Media International Australia | Journalists And Twitter: How Australian News Organisations Adapt To A New Medium | N/A | Qualitative | Journalists |
| Schultz and Sheffer | 2012 | Newspaper Research Journal | Personal Branding Still in Future For Most Newspaper Reporters | N/A | Quantitative | Journalists |
| Wetsch | 2012 | Journal Of Advertising Education | A Personal Branding Assignment Using Social Media | N/A | Qualitative | Academia (students) |
| McCorkle & McCorckle | 2012 | Marketing Education Review | Using LinkedIn in the marketing classroom: Exploratory insights and recommendations for teaching social media/networking | N/A | Qualitative | Academia (students) |
| Brandabur | 2012 | Leveraging Technology For Learning | Personal Branding Of A Teacher - An Approach Into E-Educational Environment | <PBing> usage of marketing and sociology tools in order to develop and manage somebody's public profile and professional career | Conceptual | Academia (scholars) |
| Ward & Yates | 2013 | Journal Of Service Science | Personal Branding And e-Professionalism | N/A | Conceptual | N/A |
| Parmentier et al. | 2013 | Journal of the Academy of Marketing Science | Positioning person brands in established organizational fields | <PB> set of associations that a group of people identify with a particular person | Qualitative | Models |
| Chen | 2013 | Journal Of Internet Commerce | Exploring Personal Branding on YouTube | <PBing> an art consisting of impression management and more or less strategic practices for the purpose of projecting a desired impression | Qualitative | General public |
| Karaduman | 2013 | 9th International Strategic Management Conference | The effect of social media on personal branding efforts of top level executives | <PBing> process whereby people and their careers are marked as brands <...> to create an asset and brand equity that pertains to a particular person or individual | Quantitative | CEO |
| Bendisch et al. | 2013 | European Journal Of Marketing | Fame and fortune: a conceptual model of CEO brands | <PB> an identifiable product, service, person or place, augmented in such a way that the buyer or user perceives relevant unique added values which match their needs most closely | Conceptual | CEO |
| Pihl | 2013 | Journal Of Global Fashion Marketing | In the borderland between personal and corporate brands – the case of professional bloggers | <PBing> process whereby an individual's key attributes are first identified; then, based on this assessment, a personal brand statement is constructed around this set of attributes. In the final step, a strategy for making the brand visible to the outside world is created. | Qualitative | Bloggers |
| Stanton & Stanton | 2013 | Marketing Education Review | Building "Brand Me": Creating a Personal Brand Statement | <PBing> a way for individuals to differentiate themselves by identifying their unique value proposition and communicating it effectively and consistently | Mixed methods | Academia (students) |
| Bridgen | 2014 | Journal of Media Practice | Emotional labour and the pursuit of the personal brand: Public relations practitioners' use of social media | N/A | Conceptual | PR practicioners |
| Gander | 2014 | Perspectives: Policy and Practice in Higher Education | Managing your personal brand | <PB> the clear concept that comes to mind whenever people think of us | Conceptual | N/A |
| Gioia et al. | 2014 | Research in Organizational Behavior | Image is everything Reflections on the dominance of image in modern organizational life | N/A | Conceptual | N/A |
| Baharuddin et al. | 2014 | Vision 2020 | Conceptualizing Personal Branding for Librarians | <PB> a good or service, and is a seller promise to deliver consistently a specific set of features, benefits and services to buyers | Conceptual | Librarians |
| de la Morena Taboada | 2014 | Historia y Comunicación Social | Personal Brand: concept and evolution. The influence of the press in the creation of a personal brand in the Victorian era. | <PB> a way to identify an image, a symbol or a person as something valuable, reliable and desirable | Conceptual | Celebrities |
| Garcia Montero et al. | 2014 | Historia y Comunicación Social | Self-concept and personal Brand development. Comparative analysis in international student | <PB> is the result of the process, objective of which is the identification of what is valuable, trustworthy and useful of the person | Quantitative | Academia (students) |
| Edmiston | 2014 | Marketing education review | Creating a Personal Competitive Advantage by Developing a Professional Online Presence | N/A | Mixed methods | Academia (students) |
| Hodge & Walker | 2015 | International Journal of Sport Management and Marketing | Personal branding: a perspective from the professional athlete-level-of-analysis | <PB> a public persona of an individual […] who has established their own symbolic meaning and value using their name, face, or other brand elements in the market | Qualitative | Sportsmen |
| Speed et al. | 2015 | Journal of Political Marketing | Human branding in political marketing: Applying contemporary branding thought to political parties and their leaders | Same as Close et al. (2011) | Conceptual | Politicians |
| Balbino et al. | 2015 | Revista Internacional de Relaciones Publicas | Personal Style: A strategic tool for Public Relations | <PB> an accumulation of everything that the person has already done, is doing or will do [impacting] their personal trajectory | Qualitative | Politicians |
| Philbrick & Cleveland | 2015 | Medical reference services quarterly | Personal branding: building your pathway to professional success. | <PBing> introspective process by which you define yourself professionally <PB> the combination of personal attributes, values, drivers, strengths and passions you draw from that differentiates your unique promise of value from your peers | Conceptual | N/A |
| Khedher | 2015 | The Journal of Global Business Issues | A Brand for Everyone: Guidelines for Personal Brand Managing | <PBing> planned process in which people make efforts to market themselves | Conceptual | N/A |
| Dumitri & Ciobanu | 2015 | Strategica | Personal Branding: The Marketization of Self in the Digital Landscape | <PBing> a self-centered and highly individualistic approach to self marketing <…> aimed to provide us differentiation and to make this differentiation marketable | Conceptual | N/A |
| Brooks & Anumudu | 2015 | Adult Learning | Identity Development in Personal Branding Instruction: Social Narratives and Online Brand Management in a Global Economy | <PBing> deployment of individuals’ identity narratives for career and employment purposes | Conceptual | N/A |
| Milovanovic et al. | 2015 | International Review | Personal branding through leadership | <PBing> process of identification and exploitation of strategic resources in entrepreneurial ventures | Conceptual | N/A |
| Vallas & Cummins | 2015 | Organization Studies | Personal Branding and Identity Norms in the Popular Business Press: Enterprise Culture in an Age of Precarity | N/A | Qualitative | N/A |
| Zinko & Rubin | 2015 | Journal of Management & Organization | Personal reputation and the organization | N/A | Conceptual | N/A |
| Molyneux | 2015 | Journalism | What journalists retweet: Opinion, humor, and brand development on Twitter | <PBing> direct self-promotion | Qualitative | Journalists |
| Nolan | 2015 | Public Relations Review | The impact of executive personal branding on non-profit perception and communications | <PBing> a concept that encompasses the strategies that one uses to promote one's self, both on and offine | Qualitative | CEO |
| Fetscherin | 2015 | Journal of Business Strategy | The CEO branding mix | N/A | Conceptual | CEO |
| Saleem & Iglesias | 2015 | Ideas in Marketing | Online Personal Branding In The Middle East And North America: A Comparison Of Social Capital Accumulation And Community Response | N/A | Qualitative | Bloggers |
| Manai & Holmlund | 2015 | Marketing Intelligence & Planning | Self-marketing brand skills for business students | <PBing> how an individual's sets of skills, motivations and interests are arranged, crystallized and labeled; a programmatic set of strategies for individuals to improve their chances at business success; the process by which an individual formulates and communicates his/her skills and abilities in a manner that enables him/her to stand out in a crowd and increase employment likelihood in any situation involving job hunting, developing a career and/or networking | Qualitative | Academia (students) |
| Kleppinger & Cain | 2015 | American Journal of Pharmaceutical Education | Personal Digital Branding as a Professional Asset in the Digital Age | <PB> a strategic self-marketing effort, crafted via social media platforms, which seeks to exhibit an individual's professional persona | Conceptual | Academia (students) |
| Green | 2016 | Sport, Business and Management: An International Journal | The impact of social networks in the development of a personal sports brand. | N/A | Qualitative | Sportsmen |
| Geurin-Eagleman & Burch | 2016 | Sports Management Review | Communicating via photographs: A gendered analysis of Olympic athletes' visual self-presentation on Instagram | <PB> associations one ascribes to a particular person | Qualitative | Sportsmen |
| Pera et al. | 2016 | Journal of Interactive Marketing | Who Am I? How Compelling Self-storytelling Builds Digital Personal Reputation | <PBing> capturing and promoting an individual's strengths and uniqueness to a target audience | Mixed methods | General public |
| Eagar & Dann | 2016 | European Journal of Marketing | Classifying the narrated #selfie: genre typing human-branding activity | Same as Close et al. (2011) | Qualitative | General public |
| Resnick et al. | 2016 | International Journal of Entrepreneurial Behaviour & Research | Marketing in SMEs: a "4Ps" self-branding model | <PBing> creating an identity that associates certain perceptions of you by others, which has positive benefits | Qualitative | Entrepreneurs |
| Gandini | 2016 | Marketing Theory | Digital work: Self-branding and social capital in the freelance knowledge economy | <PBing> curation of an online branded persona [combined] with the strategic management of social relationships, pointing at the acquisition of a reputational capital which may be mobilized and accessed by professionals embedded within a network of personal contacts | Qualitative | Entrepreneurs |
| Chen & Chung | 2016 | Journal of Human Resource and Sustainability Studies | How to Measure Personal Brand of a Business CEO | <PB> a representation of individualized personality traits, values, competency, and leadership ethics which effectively differentiate [an individual] and guide their decisions, enabling them to influence others deliberately | Quantitative | CEO |
| Johns & English | 2016 | Journal of Business Research | Transition of self: Repositioning the celebrity brand through social media-The case of Elizabeth Gilbert | N/A | Qualitative | Celebrities |
| Kucharska & Dąbrowski | 2016 | European Conference on Innovation & Entrepreneurship | Tacit Knowledge Sharing and Personal Branding: How to Derive Innovation From Project Teams? | <PB> gathering the capital in minds of friends and colleagues | Quantitative | Business |
| van der Land et al. | 2016 | HCI in Business, Government, and Organizations | Professional Personal Branding: Using a "Think-Aloud" Protocol to Investigate How Recruiters Judge LinkedIn Profile Pictures | <PBing> the process by which individuals attempt to control the impressions others form of them | Qualitative | Business |
| Lee & Cavanaugh | 2016 | Journal of Hospitality, Leisure, Sport & Tourism Education | Building your brand: The integration of infographic resume as student self-analysis tools and self-branding resources | <PBing> process of strategic efforts aimed at establishing favorable impressions | Conceptual | Academia (students) |
| Delisle & Parmentier | 2016 | Journal of Global Fashion Marketing | Navigating person-branding in the fashion blogosphere | N/A | Qualitative | Bloggers |
| Geurin | 2017 | Journal of Sport Management | Elite Female Athletes? Perceptions of New Media Use Relating to Their Careers: A Qualitative Analysis | N/A | Qualitative | Sportsmen |
| Tussyadiah | 2017 | Information and Communication Technologies in Tourism | Strategic self-presentation in the sharing economy: Implications for host branding | <PBing> process by which individuals differentiate themselves from the crowd by articulating their unique value proposition and leveraging it with a consistent image across different platforms to achieve their goals | Quantitative | Shared economy participants |
| Cunningham et al. | 2017 | Technology in Society | Behind the screen: Commercial sex, digital spaces and working online | N/A | Mixed methods | Sex workers |
| Rangarajan et al. | 2017 | Business Horizons | Strategic personal branding—And how it pays off | <PB> totality of impressions communicated by an individual | Qualitative | Sales executives |
| Cederberg | 2017 | Professional Psychology: Research and Practice | Personal branding for psychologists: Ethically navigating an emerging vocational trend. | <PBing> active process of synthesizing and packaging a personal brand to target customers, prospective employers, and an online network of colleagues <PB> a unique combination of various personal attributes, values, strengths, and passions that serve to differentiate value to colleagues and customers | Conceptual | Psychologists |
| Richey et al. | 2017 | Information Systems Frontiers | The Perils and Promises of Self-Disclosure on Social Media | N/A | Qualitative | Professionals |
| Evans | 2017 | Journal of Global Scholars of Marketing Science | A strategic approach to self-branding | <PBing> how we want to be perceived by employers, potential employers, clients, professional peers, and others in a way that will boost short- and long-term career prospects | Conceptual | Professionals |
| Coesemans & De Cock | 2017 | Journal of Pragmatics | Self-reference by politicians on Twitter: Strategies to adapt to 140 characters | N/A | Mixed methods | Politicians |
| Rozanova | 2017 | RUDN Journal of Sociology | Priority Value Characteristics of the Governor’s Personal Brand | <PB> combination of most significant values, creating a comprehensive symbolic image, based on image and reputation in response to the needs and interests of the target audience | Quantitative | Politicians |
| Kucharska & Confente | 2017 | Handel Wewnętrzny | Selfie and personal branding phenomena in the context of the network economy. A literature review | N/A | Conceptual | N/A |
| Ottovordemgentschenfelde | 2017 | Journalism | ‘Organizational, professional, personal’: An exploratory study of political journalists and their hybrid brand on Twitter | <PBing> deliberate actions and practices that are aimed at creating those associations  <PB> association in the minds of customers and other important constituents [which] differentiate the brand and establish <…> competitive superiority | Qualitative | Journalists |
| Hedman | 2017 | Journalism | Making the most of Twitter: How technological affordances influence Swedish journalists’ self-branding | N/A | Quantitative | Journalists |
| Brems et al. | 2017 | Digital Journalism | Personal Branding on Twitter | <PBing> distinctive presentation of a person’s character and capacity | Mixed methods | Journalists |
| Hernando & Campo | 2017 | International Journal of Arts Management | Does the Artist's Name Influence the Perceived Value of an Art Work? | <PB> a symbol or name that differentiates and influences the purchase decision, assigning the product a utilitarian and/or symbolic value | Quantitative | General public |
| Holton & Molyneux | 2017 | Journalism | Identity lost? The personal impact of brand journalism | <PBing> process of forming relationships with potential buyers, often via media as well as other communication techniques with the goal to create loyalty, an economic and emotional attachment that drives a desire to buy and/or share the product | Qualitative | General public |
| Kucharska | 2017 | Cogent Business & Management | Consumer social network brand identification and personal branding. How do social network users choose among brand sites? | <PBing> planned process in which people make efforts to market themselves | Quantitative | General public |
| Tarnovskaya | 2017 | JOURNAL OF INTERNATIONAL BUSINESS RESEARCH AND MARKETING | Reinventing Personal Branding Building a Personal Brand through Content on YouTube | N/A | Qualitative | General public |
| Schlosser et al. | 2017 | Human Resource Management | Chance Events and Executive Career Rebranding: Implications For Career Coaches and Nonprofit HRM | <PB> a reputation that others respond to and reinforce | Qualitative | Executives |
| Pagis & Ailon | 2017 | Work and Occupations | The Paradoxes of Self-Branding: An Analysis of Consultants’ Professional Web Pages | <PBing> purposeful construction of stylized self-images for self-promotion in the labor and service markets | Qualitative | Consultants |
| Dubey | 2017 | IUP Journal of Brand Management | Patanjali : Unique Brand Building and 4Ps | N/A | Qualitative | CEO |
| Abrate & Viglia | 2017 | Journal of Travel Research | Personal or Product Reputation? Optimizing Revenues in the Sharing Economy | N/A | Quantitative | Shared economy participants |
| Johnson | 2017 | International Journal of Education and Social Science | The Importance of Personal Branding in Social Media: Educating Students to Create and Manage their Personal Brand | <PBing> practice of marketing oneself to society | Conceptual | Academia (students) |
| Bergh et al. | 2017 | Critical Arts | Social Media, Permanence, and Tattooed Students: The Case for Personal, Personal Branding | <PBing> naming a product or service in order to gain an identity, develop a meaning, and project an image | Quantitative | Academia (students) |
| Jones & Leverenz | 2017 | International Journal of ePortfolio | Building Personal Brands with Digital Storytelling ePortfolios | N/A | Conceptual | Academia (students) |
| Caro | 2017 | Area Abierta | The Discourse of Self-Promotion and Authenticity in Social Networking Sites: Personal Branding and Microcelebrity | N/A | Qualitative | General public |
| Jaring & Bäck | 2017 | Technology Innovation Management Review | How Researchers Use Social Media to Promote their Research and Network with Industry | N/A | Qualitative | Academia (scholars) |
| López-Meri & Casero-Ripollés | 2017 | Revista Mediterránea de Comunicación | Journalists' strategies to build personal brand on Twitter: positioning, content curation, personalization and specialisation | <PBing> manner to present one's character, distinctive capabilities and skills | Qualitative | Journalists |
| Hanush & Bruns | 2017 | Digital Journalism | Journalistic Branding on Twitter | <PBing> same as Gandini (2016) | Quantitative | Journalists |
| Amoako & Okpattah | 2018 | Technology in Society | Unleashing salesforce performance: The impacts of personal branding and technology in an emerging market | <PBing> discovery, understanding and marketing of an individual's unique attributes | Quantitative | Sales executives |
| Vallas & Christin | 2018 | Work and Occupations | Work and Identity in an Era of Precarious Employment: How Workers Respond to “Personal Branding” Discourse | N/A | Qualitative | Journalists |
